# Sensory Conflict Disrupts Activity of the *Drosophila* Circadian Network

**DOI:** 10.1016/j.celrep.2016.10.029

**Published:** 2016-11-08

**Authors:** Ross E.F. Harper, Peter Dayan, Joerg T. Albert, Ralf Stanewsky

**Affiliations:** 1 Centre for Mathematics, Physics and Engineering in the Life Sciences and Experimental Biology (CoMPLEX), University College London, London WC1E 6BT, UK; 2 Ear Institute, University College London, London WC1X 8EE, UK; 3 Gatsby Computational Neuroscience Unit, University College London, London W1T 4JG, UK; 4 Department of Cell and Developmental Biology, University College London, London WC1E 6BT, UK

## Abstract

Periodic changes in light and temperature synchronize the *Drosophila* circadian clock, but the question of how the fly brain integrates these two input pathways to set circadian time remains unanswered. We explore multisensory cue combination by testing the resilience of the circadian network to conflicting environmental inputs. We show that misaligned light and temperature cycles can lead to dramatic changes in the daily locomotor activities of wild-type flies during and after exposure to sensory conflict. This altered behavior is associated with a drastic reduction in the amplitude of PERIOD (PER) oscillations in brain clock neurons and desynchronization between light- and temperature-sensitive neuronal subgroups. The behavioral disruption depends heavily on the phase relationship between light and temperature signals. Our results represent a systematic quantification of multisensory integration in the *Drosophila* circadian system and lend further support to the view of the clock as a network of coupled oscillatory subunits.

## Highlights

• Conflicting light and temperature cycles lead to abnormal, plateau-like locomotor behavior
• Plateau-like behavior is accompanied by a collapse of the molecular circadian clock
• Temperature cues dominate during small light and temperature misalignments
• Light cues dominate during large light and temperature misalignments

## Graphical Abstract

**Figure:**
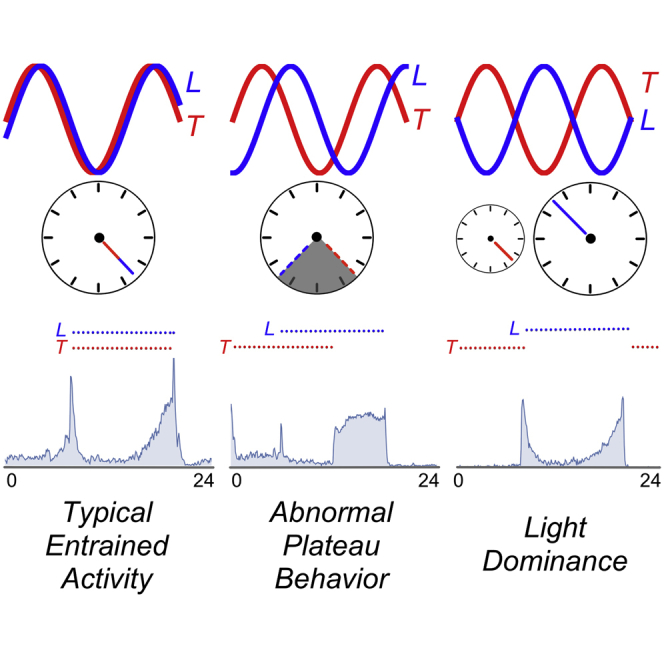

## Teaser

Circadian clocks allow bodily functions to occur at optimal times of day and need to be synchronized with the local environment. Harper et al. examine how circadian clocks respond to situations of sensory conflict and show that in *Drosophila,* intermediate (5–7 hr) offsets between light and temperature cycles lead to an altered activity profile, underpinned by a drastically compromised molecular clock.

## Introduction

Circadian networks generate endogenous rhythms that optimize the behavior of organisms for a periodic environment. However, environmental fluctuations are themselves intrinsically variable, changing across seasons and latitudes. A reliable circadian pacemaker must therefore possess the capacity to synchronize its oscillations to periodic environments without being disturbed by short and sporadic changes, as exist under natural conditions. In the fruit fly, *Drosophila melanogaster*, the two most potent clock-resetting signals, or Zeitgebers (ZG), are light-dark (LD) and temperature cycles (TCs). Individually and together, these two sensory modalities can entrain locomotor activity rhythms as well as molecular rhythms in clock cell groups throughout the fly (Dubruille and Emery, 2008, Glaser and Stanewsky, 2005, Plautz, 1997, Sehadova et al., 2009, Wheeler et al., 1993, Yoshii et al., 2009, Zerr et al., 1990). This poses a question of sensory integration: how are different, and potentially conflicting, sources of information integrated by the clock to compute circadian time and produce a coherent behavioral output?

Coordinated circadian behavior in *Drosophila* emerges from the concerted activity of a network of ∼150 clock neurons located in the central nervous system, which are endowed with the intracellular capacity for circadian rhythmicity (Peschel and Helfrich-Förster, 2011). A traditional view of the clock highlights the small Pigment Dispersing Factor (PDF)-positive lateral ventral neurons (s-LN_v_s) as autonomous pacemakers, which impose rhythmicity on a more passive remainder of the network (Renn et al., 1999). The reality, however, is likely to be more complicated. Indeed, experimental conditions heavily influence both the supposed identity of these clock “masters” and the precise network hierarchy reported (Helfrich-Förster et al., 2007).

Laboratory conditions typically treat ZGs in a singular manner; circadian networks, however, operate subject to multisensory challenges. This concept has been embraced by a small number of previous studies, which form the foundation of our work (Currie et al., 2009, Miyasako et al., 2007, Yoshii et al., 2010). In one, LD and TCs were misaligned by 12 hr (Yoshii et al., 2010)—an antiphasic relationship that represents the largest possible disparity between two 24-hr environmental oscillators. During this extreme sensory conflict, activity rhythms of wild-type flies entrain preferentially to the light stimulus, leading to the conclusion that this cue is dominant (a prevailing view in the field). However, in a similar study investigating antiphasic LD:TC, temperature was found to have a more substantial circadian effect, advancing the onset of evening locomotor activity (Currie et al., 2009). Moreover, field studies exploiting naturalistic environmental fluctuations demonstrate a more prominent role of temperature in locomotor entrainment (Vanin et al., 2012). The situation thus remains unclear. The analysis of one single signal disparity is insufficient to fully probe the possible coupling at play in the *Drosophila* circadian system.

In another study, a smaller degree of environmental misalignment was implemented using a 6-hr advance of TC relative to LD (Miyasako et al., 2007). However, the comparatively small amplitude TC (20:25°C), for what is regarded as the weaker of the two ZGs in *Drosophila* (Yoshii et al., 2010) is likely to have been insufficient to distinguish subtle signal averaging effects from background noise, especially given the much larger temperature ranges found in nature (Vanin et al., 2012). Again, this might explain the relatively undisturbed light-aligned locomotor activity observed under these specific conditions.

To better understand the effect of environmental phase relationships on circadian clock function, we assessed circadian locomotor behavior during misaligned LD and TC using finer gradations of sensory conflict and greater diurnal fluctuations in both cues. Furthermore, we compared wild-type flies to *cry*-null mutants, removing the key contribution made by the circadian photoreceptor Cryptochrome (CRY) to light entrainment of the clock (Stanewsky et al., 1998). We hypothesized that any effect of multisensory integration would be markedly diminished in *cry* mutants, owing to a reduced weight of the light-dependent input pathway and relative enhancement of the temperature cue (Gentile et al., 2013).

## Results

### Sensory Conflict Disrupts Normal Daily Locomotor Activity

While recent studies have aimed to generate more naturalistic environmental transitions (e.g., Vanin et al., 2012), our study of the mechanistic bases of ZG integration requires the establishment of deliberately *unnatural* experimental conditions. Note that we refer to cue misalignment as the absolute distance, in hours (*delta* time, or Δt), between onset/offset of two cyclic 12-hr:12-hr signals. For example, Δt_L,T_ = 3 hr denotes that light onset/offset occurs 3 hr after temperature.

**Figure 1:**
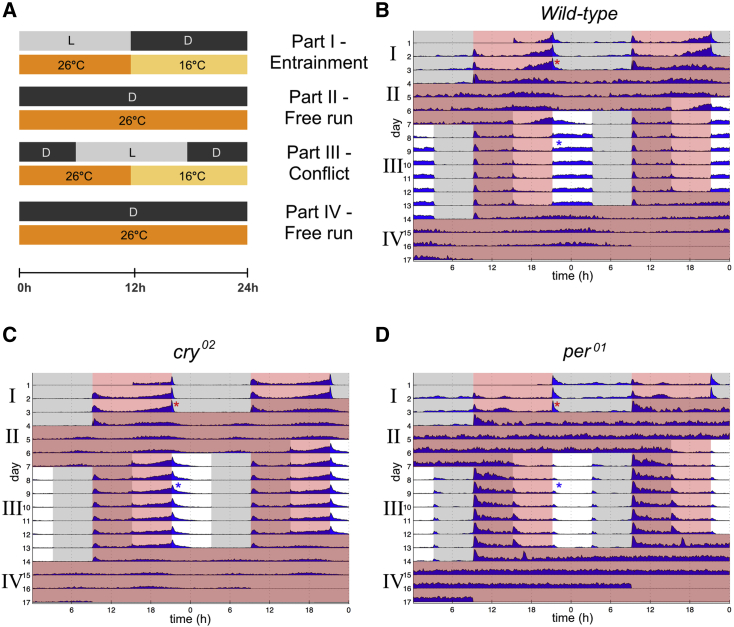
Locomotor Behavior during Sensory Conflict (A) Experimental regime in which environmental conditions followed 3 days of 12-hr:12-hr LD and TC (16:26°C) in-phase (I), 3 days of free run in DD at 26°C (II), 7 days of out-of-phase 12-hr:12-hr LD and TC (16:26°C) via 6-hr delay of LD (III), followed by 3 days of free run in DD at 26°C (IV). (B–D) Average actograms of wild-type (B) (n=46), *cry*^*02*^ (C) (n=44), and *per*^*01*^ (D) (n=32). Red asterisk denotes representative evening behavior in part I; blue asterisk denotes representative pseudo-evening behavior in part III. Clock-less *per*^*01*^flies show only brief startle responses to the sudden environmental changes and otherwise display arrhythmic behavior (C). See Figure S1 and S2 for individual fly data and genetic controls.

Wild-type flies (Canton S) and *cry*-null mutants (*cry*^*02*^) were subjected to an environmental regime comprising aligned LD:TC (part I, Δt_L,T_ = 0 hr), followed by a 6-hr delay of LD with respect to TC (part III, Δt_L,T_ = 6 hr), interspersed or followed by free running conditions to assess stability of endogenous rhythms (part II and part IV, outlined in Figure 1A). As is standard practice for observing endogenous activity rhythms, free running conditions comprised constant darkness and constant warmth (26°C—*Drosophila*’s preferred ambient temperature [Sayeed and Benzer, 1996]) to mitigate any negative masking effect of cold temperatures on overall activity levels.

In part I, locomotor behavior in wild-type and *cry*^*02*^ flies both displayed a characteristic bimodal profile, showing an evening peak of activity that coincided with the end of photo/thermo-phase (Figures 1B and 1C). These entrained rhythms persisted in free-running conditions (part II). In part III, a 6-hr misalignment between LD and TC was introduced via a 6-hr delay of LD relative to part I (leaving TC unchanged). Under sensory conflict, circadian locomotor behavior in wild-type flies was drastically altered, exhibiting a plateau of sustained activity between temperature offset and light offset, bordered by periods of inactivity (Figure 1B). The activity pattern continued for the duration of the conflict and was also seen at the level of individual flies, and across multiple repeats (Figures S1 and S2E). A key facet of this activity pattern is the absence of any evening anticipation to either the light or the temperature cue. For ease, we refer to this abnormal locomotor behavior as “plateau” (P) behavior. Importantly, P behavior depends on a functional clock as it cannot be observed in *per*^*01*^ mutants (Figure 1D). That P behavior is not merely induced by masking is also apparent from comparing the free running behavior in part IV with that in part II (Figures 1B and S2E).

The P behavior observed in wild-type flies was not present in *cry*^*02*^ mutants during conflict conditions, which instead displayed the typical ramping increase of activity, peaking at temperature offset (Figure 1C). This suggests these flies predominantly entrained to TC. However, we do note the behavioral profile is slightly altered from that in part I, for instance, including an extended period of activity after temperature offset. The conflicting regime (and therefore the periodic presence of light) appears to have had some effect, albeit greatly reduced, on the behavior of *cry*^*02*^ mutants. This observation is consistent with the existence of *cry*-independent light entrainment pathways (Yoshii et al., 2015).

To test whether the absence of P behavior in *cry*^02^ mutants was indeed due to the absence of CRY, we rescued *cry* expression in all clock cells or all clock neurons (*tim-gal4/* and *Clk856-gal4/UAS-cry;cry*^*b*^*/cry*^*01*^, respectively). Rescue flies displayed activity rhythms that more closely resembled the wild-type than the *cry*^*02*^ pattern—inactive prior to temperature offset, with a bout of activity between temperature and light offset (Figure S2). These data suggest that it is indeed the integration of two potent, yet conflicting input signals to the clock—one photic and the other non-photic—that underlies the abnormal behavioral output observed in sensory conflict.

### Sensory Conflict Disrupts Endogenous Oscillations in the Central Clock Network

Cytological staining for clock gene products has revealed the location of the central circadian network in *Drosophila* (Ewer et al., 1992, Frisch et al., 1994, Zerr et al., 1990), which can be further classified into seven distinct cell groups: small and large ventral lateral neurons (s-LN_v_, l-LN_v_), dorsal lateral neurons (LN_d_), the first, second, and third dorsal neuron groups (DN_1_, DN_2_, and DN_3_) and the lateral posterior neurons (LPNs) (Nitabach and Taghert, 2008). While there are likely to be additional subdivisions within the network (Peschel and Helfrich-Förster, 2011), our study of multisensory processing in the fly brain adopted the prevailing, and well-supported, network architecture. Indeed, it has been shown previously that the molecular rhythms of clock neurons expressing CRY appear to entrain preferentially to light, whereas the CRY-negative DN_2_ and LPN subgroups entrain preferentially to temperature in 12-hr conflicting LD:TC (Yoshii et al., 2010).

To examine the molecular and neuronal substrates of the pronounced P behavior, we carried out antibody staining for the clock protein PERIOD (PER) in the *Drosophila* brain during 6-hr misaligned LD:TC. PER immunostaining of clock neurons was performed at four time points evenly spaced across 24 hr (ZT3, ZT9, ZT15, and ZT21) for both part I and part III of the experimental regime. In flies that have entrained to a given ZG, maximum and minimum staining intensity is expected at ZT21 and ZT9 respectively (Yoshii et al., 2009) (note that during sensory conflict ZT_*L*_ and ZT_*T*_ refer to ZT specified by light and temperature, respectively).

**Figure 2:**
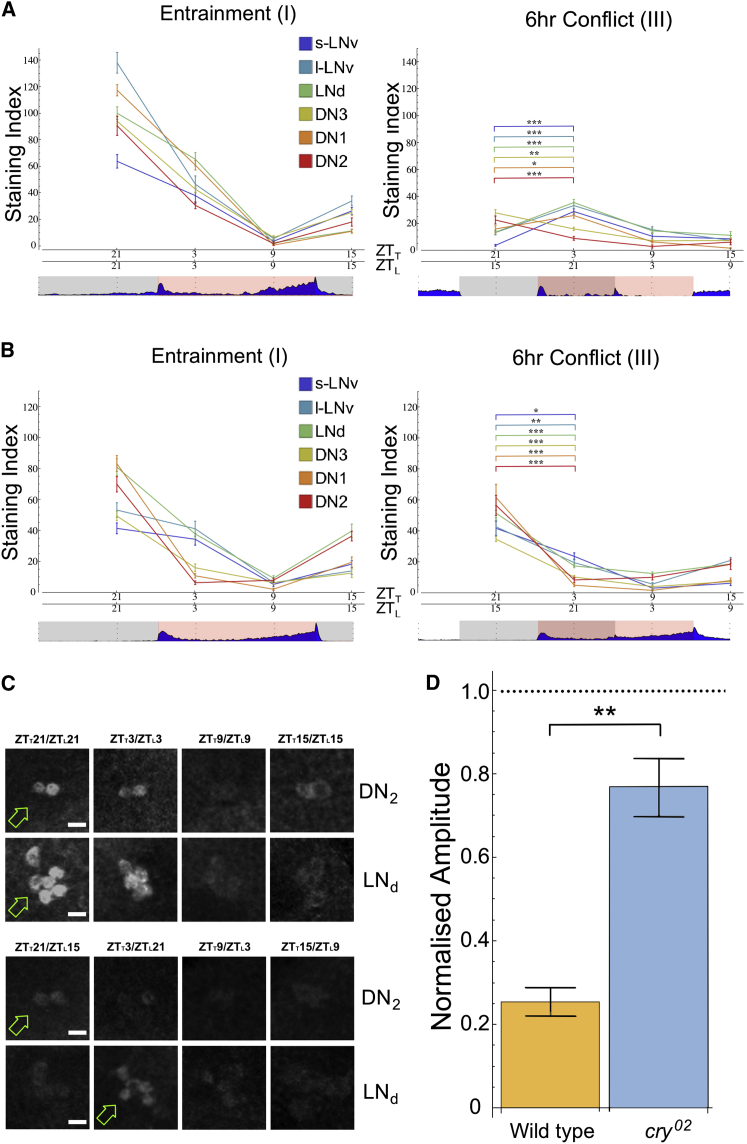
Central Clock Molecular Rhythms during Sensory Conflict (A and B) PER immunostaining of wild-type (A) and *cry*^*02*^ (B) brains during entrainment (left: TC and LD in sync) and 6-hr conflict (Right) conditions. One-way ANOVA reveals a significant effect of ZT on PER staining intensity under in-phase and out-of-phase conditions in both genotypes (p < 1 × 10^−7^ in all clock neuronal groups). During 6-hr conflict, t test reveals significant differences between the first two time points plotted for all neuronal subgroups in wild-type and *cry*^*02*^. Dissociation in peak staining between different neuronal groups occurred in wild-type, but not in *cry*^*02*^ (see also Table S1). (C) PER staining in the DN_2_ and LN_d_ cell groups in wild-type brains during entrainment (top) and 6-hr conflict (bottom) conditions. Scale bar, 5 μm. Green arrows mark maximum staining for each cell group. (D) Average amplitude of neuronal subgroup oscillations during sensory conflict (part III) divided by that during entrainment conditions (part I) in wild-type and *cry*^*02*^. A score of 1 denotes no change between conditions. All error bars represent SEM (p < 0.01^∗^p < 0.001^∗∗^p < 0.0001^∗∗∗^).

During in-phase LD:TC (part I), wild-type flies showed the expected strong PER oscillations in all neuronal subgroups with a peak at ZT21 and a trough at ZT9 (Figures 2A, left, and 2C, top). In *cry*^*02*^ mutants, PER cycled with the same phase, but with lower amplitude (Figure 2B, left), consistent with previous findings that light and temperature synergistically entrain molecular rhythms (Yoshii et al., 2009).

By contrast, during conflict, we observed a striking collapse in the amplitude of PER oscillations for all neuronal subgroups in wild-type flies (Figures 2A, right, 2C, bottom, and 2D). Furthermore, inspecting the residual low-amplitude PER oscillations, there appeared to be a clear shift in the peak of the s-LN_v_, l-LN_v_, LN_d_, and DN_1_ to ZT_*L*_21, suggesting at least partial entrainment of these neurons to LD. In contrast, the CRY-negative DN_2_ and DN_3_ remained phase-locked to TC, displaying peak PER expression at ZT_*T*_21. We did not notice any obvious phase heterogeneity within each neuronal subgroup (see, for example, the DN_2_ and LN_d_ in Figure 4C). In *cry*^*02*^ mutants under conflict conditions, molecular rhythms remained comparable to part I (Figures 2B, right, 2D). This echoes our behavioral findings, suggesting that the altered molecular rhythms observed in wild-type flies result from the integration of conflicting inputs to the clock network, and that such conflicts can be avoided by weakening one of the input pathways, as in *cry*^*02*^ mutants.

### Sustained Effects of Sensory Conflict on the Circadian Clock

**Figure 3:**
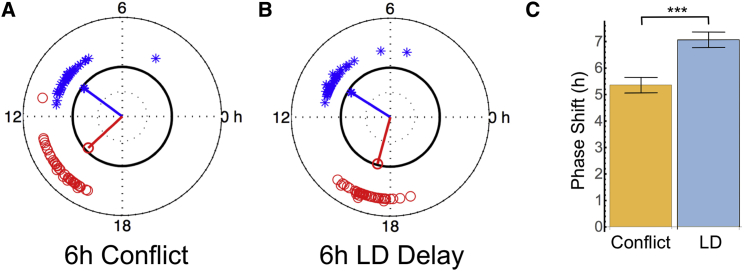
Sustained Effects of Sensory Conflict on Circadian Clock Phase Comparison of the activity peaks during the free-running parts of the experiment (parts II and IV) preceding and following exposure to (A) 6-hr delayed sensory conflict (n = 38, *phase difference* = 5.3 hr, p < 0.001) or (B) 6-hr delayed LD cycle at constant 26°C (n = 46, *phase difference* = 7.1 hr, p < 0.001). Crosses show mean phase of each fly across the first 2 days of free run. Blue shows part II; red shows part IV. Circular statistics as used in Levine et al. (2002a). (C) Bar chart showing magnitude of phase shift between part II and IV in experimental groups (A) and (B). Error bars show SD. p < 1 × 10^–7^.

Considering the drastic effects of sensory conflict on behavior and molecular clock oscillations, one would expect alterations to the underlying state of the circadian clock. This should manifest itself during constant conditions. We therefore analyzed the consequences of sensory conflict (part III) on the final free run section (part IV). We compared overall rhythmicity and peak phase during free run, with that of control flies that had not experienced sensory conflict. These control flies were initially exposed to the identical in-phase LD:TC and free-running conditions (part I and part II), before being subjected to a 6-hr delayed LD cycle at constant 26°C (part III, Δt_L_ = 6 hr) and subsequent release into the final free run (part IV, constant darkness [DD] at 26°C) (Figure S2F). While we did not observe any effects on overall rhythmicity or period length (Table S2), we did notice an advance of the activity peak in flies experiencing sensory conflict compared to those that were shifted with light at constant temperature (Figures 3A, S2E, S2F, and S3). To quantify this apparent effect of the (un-shifted) temperature cue, we determined the magnitude of the phase difference between activity rhythms in free run part II and part IV for sensory conflict and control flies using circular phase analysis (Levine et al., 2002a, Experimental Procedures). As expected, both groups displayed almost identically phased activity peaks during part II (2.4 and 2.2 hr before light and temperature onset in part I, respectively). In contrast, in the free-run (part IV) following sensory conflict, peak activity was delayed by 5.3 hr, while the peak of control flies was delayed by 7.1 hr (Figure 3). Thus, exposure to conflicting ZGs diminished the degree of activity phase shift by almost 2 hr. This observation is consistent with theoretical considerations of the clock as coupled oscillatory subunits, which predict that the resulting equilibrium phase following conflicting input is some weighted average of the two inputs. This would act to reduce the degree of phase shift compared to synchronization with the 6-hr delayed LD alone.

### Robustness of the Clock Network to Conflicting Inputs

A recent study by Yao and Shafer (2014) suggests that the *Drosophila* central clock network is resilient to period discrepancies between neuronal subgroups, such as PDF-negative and PDF-positive neurons. Indeed, it was shown that coherent activity rhythms could still be generated, provided the period length mismatch between the cell groups was less than ∼2.5 hr. Having shown that 6-hr-misaligned LD:TC generates P activity patterns (Figure 1B) associated with a severe collapse of PER oscillations in all clock cell groups, and phase differences between light- and temperature-sensitive clock subgroups (Figures 2A and 2D), we went on to explore the consequences of other degrees of sensory conflict for circadian locomotor behavior. Adapting part III of the experimental regime, we conducted a systematic behavioral analysis investigating the effect of varying the magnitude of the LD delay.

**Figure 4:**
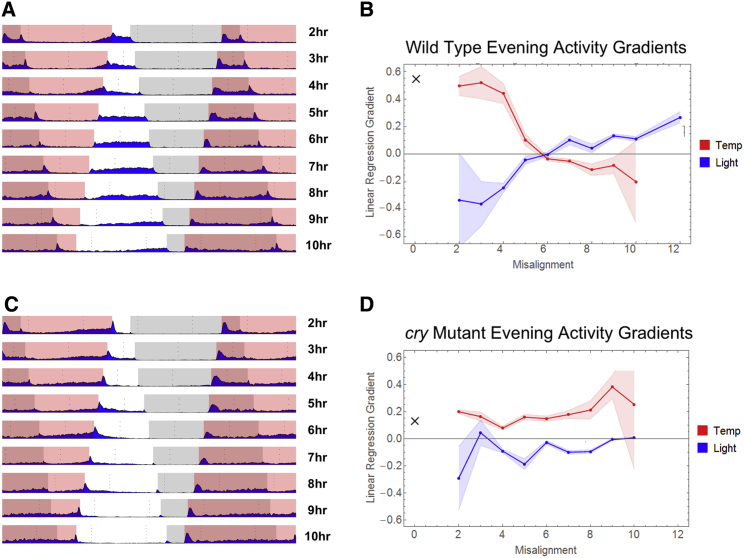
Behavioral Responses to Different Light and Temperature Phase Relationships Varying degrees of LD:TC misalignments in wild-type (A and B) and *cry*^*02*^ flies (C and D) (45 ≤ n ≤ 65). (A and C) Representative days of locomotor behavior taken from average actograms during conflict conditions after activity rhythms had stabilized (part III, days 5–6). (C and D) Gradients of linear regression fit to the period of activity preceding light and temperature cutoffs. Shaded regions denote 95% confidence intervals. Black cross indicates gradient of evening activity during corresponding in-phase condition (see also Figure S4).

When LD:TC misalignment was less than 4 hr, wild-type flies displayed anticipatory behavior and peak activity at the end of thermo-phase, thus appearing to primarily follow the temperature signal (Figure 4A). However, activity persisted after temperature offset into the lights-on phase, suggesting some effect of light on circadian locomotor behavior (reminiscent of that observed in *cry*^*02*^ mutants during 6-hr conflict). Fully fledged P behavior emerged at 5- to 7-hr misalignments, with an absence of conventional entrainment to either signal. As the disparity between LD:TC exceeded 7-hr misalignment, P behavior gradually decayed, with a discernible peak of activity observed at light offset during 10-hr misaligned conditions. These results go some way toward explaining previous observations made in antiphasic (i.e., Δt_L,T_ = 12 hr) light and temperature (Yoshii et al., 2010)—only during very large sensory conflicts is light the dominant ZG.

We quantified these observations by assessing the gradients of the gradual increase in locomotor behavior that arises toward the offset of ZGs during entrainment. When LD and TC are synchronized, the gradient is positive, consistent with evening anticipation. In our misaligned conditions, the two separate gradients associated with light and temperature offset can be used to gauge the disruption caused (see Experimental Procedures and Figure S4). The progressive change in these gradients for temperature and light with misalignment is evident in Figure 4B. P behavior occurs when both gradients approach zero. Entrainment to TC for smaller misalignments, and to LD for the largest misalignments, is also evident from the plot.

In contrast to wild-type flies, the activity rhythms of *cry*^*02*^ mutants remain largely entrained to TC, independent of the magnitude of the sensory conflict (Figure 4C). This unwavering temperature preference is again illustrated numerically by the fact that temperature evening gradients remain more positive than light evening gradients for all LD:TC misalignments (Figure 4D).

## Discussion

Circadian research in *Drosophila melanogaster* has traditionally treated light and temperature separately. However, clock networks evolved to orchestrate behavior within multisensory environments. Recent studies suggest the existence of multiple independent oscillatory subunits within the fly central clock, each capable of driving activity patterns (Yao and Shafer, 2014). Such distributed architectures tend to exhibit cooperation and/or competition. We here present a systematic and quantitative exploration of the behavioral and molecular effects of conflicting (light/temperature) entrainment regimes on the circadian system. Our paradigm offers a novel route to decompose the circadian network and our findings demonstrate that sensory conflict can—under specific conditions—cause dramatic disruptions to clock output, which have not been reported before.

Although light does indeed dominate temperature for maximal misalignments, smaller delays of LD relative to TC lead to evening activity rhythms in wild-type flies that are predominantly entrained to the temperature cue. These observations are in line with previous reports of temperature also being the critical parameter for morning activity onset in natural conditions (Vanin et al., 2012). Our findings indicate a higher biological relevance for temperature effects on daily behavioral rhythms than previously appreciated. Furthermore, with larger delays of 5–7 hr, typical evening peaks of activity broke down giving way to an abnormal locomotor pattern, which we here refer to as plateau (P) behavior. This P behavior is associated with a drastic reduction in the amplitude of molecular rhythms, as well as dissociation between clock neuronal groups. Importantly, 6-hr sensory conflict also reduced the degree of phase shift compared to that induced by 6-hr delay of light alone, demonstrating that sensory conflict alters the state of the circadian oscillator (Figure 3). It was only during even larger misalignments of 8- to 10-hr that we saw a restoration of more typical evening activity peaks and a reversal of cue preference back to the light signal (cf. Yoshii et al., 2010). Together, these results emphasize the context-dependent nature of ZG dominance. The *Drosophila* circadian system, it appears, is able to generate “wild-type-like” behavioral rhythms only for a limited range of light-temperature phase relationships, i.e., either very small or very large misalignments; intermediate conflicts, however, are not easily accommodated by the clock network.

Throughout our investigation, we have maintained a phase-agnostic approach to our experimental interpretations. It remains unclear how the phase of environmental oscillatory signals translate to circadian phase extracted by the clock. Indeed, temperature typically lags behind light under natural conditions (Boothroyd et al., 2007, Vanin et al., 2012), suggesting that Δt_L,T_ = 0 hr might not necessarily represent “in-phase” signals as far as the clock is concerned. Pending deeper understanding, we must only treat phase relationships between light and temperature in a relativistic manner. Thus, the coincidence of photo- and thermo-phases should be thought of as an arbitrary reference point (admittedly, one that has been used frequently in the field).

From a mechanistic viewpoint, our molecular data reveal a striking effect of sensory conflict, as 6-hr LD:TC misalignments lead to a drastic reduction in the amplitude of molecular rhythms in all clock neurons. The phase of the remaining low-amplitude oscillations appears largely consistent with that reported previously (Yoshii et al., 2010), revealing a temperature preference of the *cry*-negative DN_2_ in wild-type flies. Curiously, residual PER rhythms in the DN_3_ also align with TC during sensory conflict. This finding, which might be linked to our particular experimental conditions, has not been reported previously—in 12-hr conflict conditions, PER rhythms in DN_3_ preferentially entrain to light (Yoshii et al., 2010). Our results do, however, resemble TIM cycling reported previously during sensory conflict (Miyasako et al., 2007), suggesting a temperature-sensitive property of the DN_3_.

At its core, the clock network must perform multisensory integration (MSI). Bayesian methods offer a powerful way to analyze MSI, and, in the context of our results, bring to the fore two key considerations: the relative strengths of different signals; and the possibility that the signals might have different, as opposed to the same, underlying causes.

In the Bayesian characterization of timekeeping, there is a hidden or latent variable (here, the true time of day) whose values are associated with possibly noisy observations (fluctuating light and temperature signals). Different sources of an observation are integrated with different weights of influence according to their respective reliabilities. Weak periodic fluctuations in a source cue provide little reliable evidence about the time of day and so exert little effect over the estimate. This might explain why Miyasako et al. (2007) did not observe P behavior using small fluctuations in the temperature cue during conflict with LD cycles. It would be interesting to investigate whether flies are able *to learn* the reliability of different sources of input and adjust their relative weights accordingly.

Bayesian treatments of MSI also acknowledge the possibility that highly discrepant signals are unlikely to come from the same underlying value of the latent variable (Körding et al., 2007). Depending on the circumstance, inference could then reject one of the signals as being just noise; or it could infer that there is more than one underlying latent variable. In these cases, the smaller the disparity between the signals, the readier inference will be to integrate them. This could explain why aberrant P behavior only arose at conflicts of ∼5–7 hr—sufficiently large to disrupt integration, but too small to lead to segregation.

In the case of segregation, the two possibilities have different implications. Rejecting sources as being noise is a choice that itself involves assessments of relative reliabilities, and prior biases. For equally strong sources, prior bias would dominate—which might perhaps favor light. This would be consistent with the observation that the 12-hr LD:TC misalignment used by Yoshii et al. (2010) led to dominance of light entrainment, without substantial behavioral disruption. The second possibility in our case is that two different times of day are inferred, one each associated with light and temperature. This might explain our observed dissociation of distinct populations of clock neurons. Indeed, there may be physiological activities required to occur at certain temperatures, even if at what might be inappropriate light-defined times. This separation could further extend to the peripheral clock network, in which the temperature cue has been shown to have a prominent role in entrainment (Sehadova et al., 2009). It would certainly be intriguing to explore the response of these peripheral clocks to sensory conflict.

### Conclusion

Robustness toward a range of variable, and potentially conflicting, inputs is a beneficial property for any sensory network. We show that phase discrepancies between clock neurons can result from sensory conflict, and that in these conditions, the fly clock resists some, but embraces other, misalignments. Network robustness offers obvious advantages in itself, but possible benefits extend beyond this. Resilience might also imply plasticity, allowing different clock cell groups to exhibit autonomy under different conditions, truly optimizing behavior for particular environmental features. Moreover, in nature, the phase relationship between light and temperature might also provide valuable circannual information to the network.

Building on previous studies, we focused on the interplay between light and temperature in *Drosophila.* Our findings, however, are not restricted to these two sensory entrainment pathways, nor are they restricted to the fly. Links between human circadian clock function (and dysfunction) and mental disorders have been made repeatedly, but the directions of the underlying causalities are still unclear (Roenneberg and Merrow, 2016). Most intriguing in this regard is the suggestion that the associations between psychiatric pathologies and the clock partly involve behavioral habits, which alter an individual’s exposure to different ZGs (Adan et al., 2012). A more thorough study of multisensory processing in the circadian system, and possible conflicts that can arise therein, therefore stands not only to increase our understanding of the computation of time, but also to enable novel approaches in the treatment, and prevention, of mental disorders.

Other cues, such as mechanical (Simoni et al., 2014) and social (Levine et al., 2002b) ones, have been shown capable of entraining the fruit fly’s circadian clock. The case of mechanosensory clock input is particularly interesting as proprioceptive feedback from an individual’s own locomotor behavior may in fact contribute back to clock entrainment, blurring the boundaries between network output and input. We look forward to future work further disentangling the complex nature of multisensory processing in biological time-keeping systems.

## Experimental Procedures

### Activity Monitoring

Locomotor activity rhythms were recorded automatically using the *Drosophila* Activity Monitoring (DAM) system (Trikinetics) as previously described (Glaser and Stanewsky, 2005). See Supplemental Experimental Procedures.

### Data Analysis

Raw activity data were scanned using DAM File Scan software and saved into txt files. All analyses were carried out using the MATLAB Flytoolbox library (Levine et al., 2002a) and Wolfram Mathematica. See Supplemental Experimental Procedures.

### Immunostaining and Quantification

Flies were collected at four time points during the in-phase and out-of-phase conditions (corresponding to ZT3, ZT9, ZT15, and ZT21 of the in-phase condition), and brains were subsequently incubated with PER antibodies (see Supplemental Experimental Procedures). Quantification of PER signals was conducted without discrimination of sub-cellular localization using ImageJ, as described previously (Rieger et al., 2006). PDF staining served as a useful neuroanatomical marker to distinguish between LN_v_ and other clock neuronal groups. Statistical tests, including t test and ANOVA, were conducted in Mathematica.

## Author Contributions

R.E.F.H. conducted the experiments and analysis. R.E.F.H., P.D., J.T.A., and R.S. designed the experiments and authored the paper. J.T.A. is the corresponding author for circadian, computational, and conceptual questions, and R.S. is the corresponding author for circadian, experimental, and molecular issues.

## Supplemental Information

Document S1. Supplemental Experimental Procedures, Figures S1–S4, and Tables S1 and S2

Document S2. Article plus Supplemental Information
