# Supplementary material for: Sensory Conflict Disrupts Activity of the Drosophila Circadian Network
Source: Cell Rep. 2016 Nov 8;17(7):1711–8. doi: 10.1016/j.celrep.2016.10.029 (PMC5120367; doi:10.1016/j.celrep.2016.10.029)
Supplement: Document S1. Supplemental Experimental Procedures, Figures S1–S4, and Tables S1 and S2 [file mmc1.pdf]

Cell Reports, Volume *17*

Supplemental Information

Sensory Conflict Disrupts Activity of the *Drosophila* Circadian Network

Ross E.F. Harper, Peter Dayan, Joerg T. Albert, and Ralf Stanewsky

Figure S1 (Related to Figure 1)

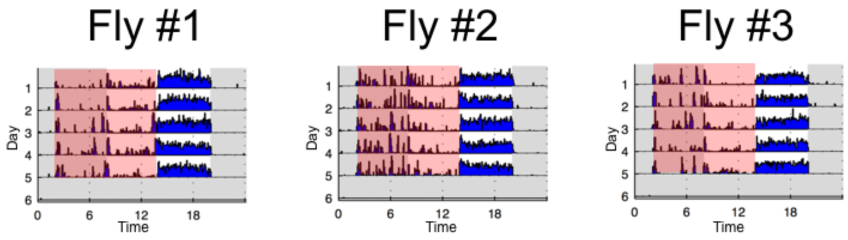

Figure S1. Novel P Behaviour Observed in Individual Flies
Three representative activity traces taken from individual CantonS flies during 6h misalignment of LD and TC (16:26°C). Red filter denotes warm phase; white background denotes light phase.

Figure S2 (Related to Figures 1 and 3)

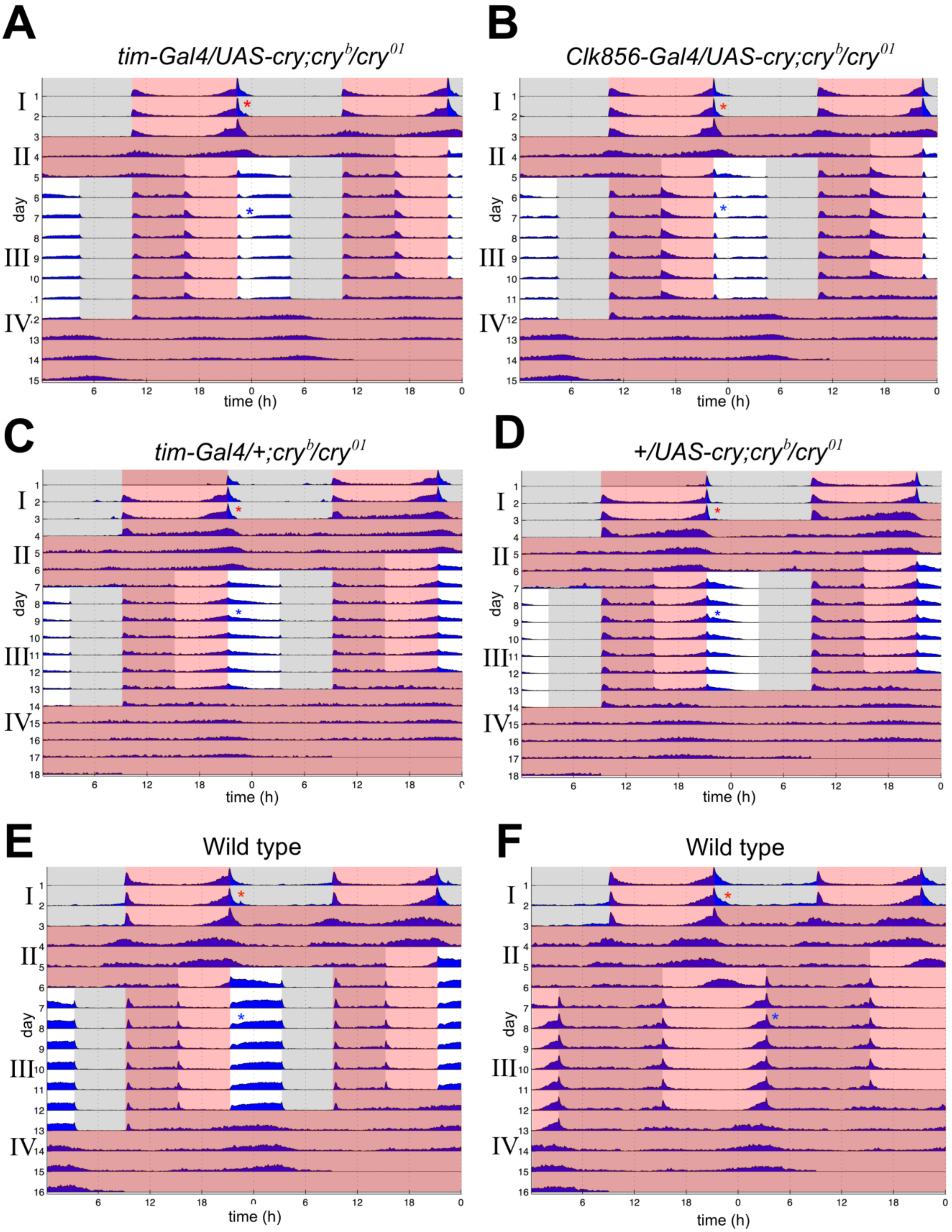

Figure S2. Circadian Locomotor Behavior During Sensory Conflict

Average actograms of (A) *cry* mutant rescue using a tim-Gal4 driver ($n = 32$), (B) *cry* mutant rescue using a *Clk856-Gal4* driver($n = 33$), (C*) tim-Gal4/+;cry^{b}/cry^{01}* control ($n = 12$), (D) *+/UAS-cry;cry^{b}/cry^{01}*control ($n = 21$), (E) Wild type repeat ($n = 38$), and (F) wild type during LD jet lag experiment (Part III comprises 6h delay of LD relative to Part I, at constant 26°C) ($n = 46$). Environmental conditions outlined in Figure 1. Red filter shows warm phase; white background shows light phase. Red asterisk denotes representative evening behaviour in Part I; blue asterisk denotes representative pseudo-evening behaviour in Part III.

Figure S3 (Related to Figure 3 and S2 E, F)

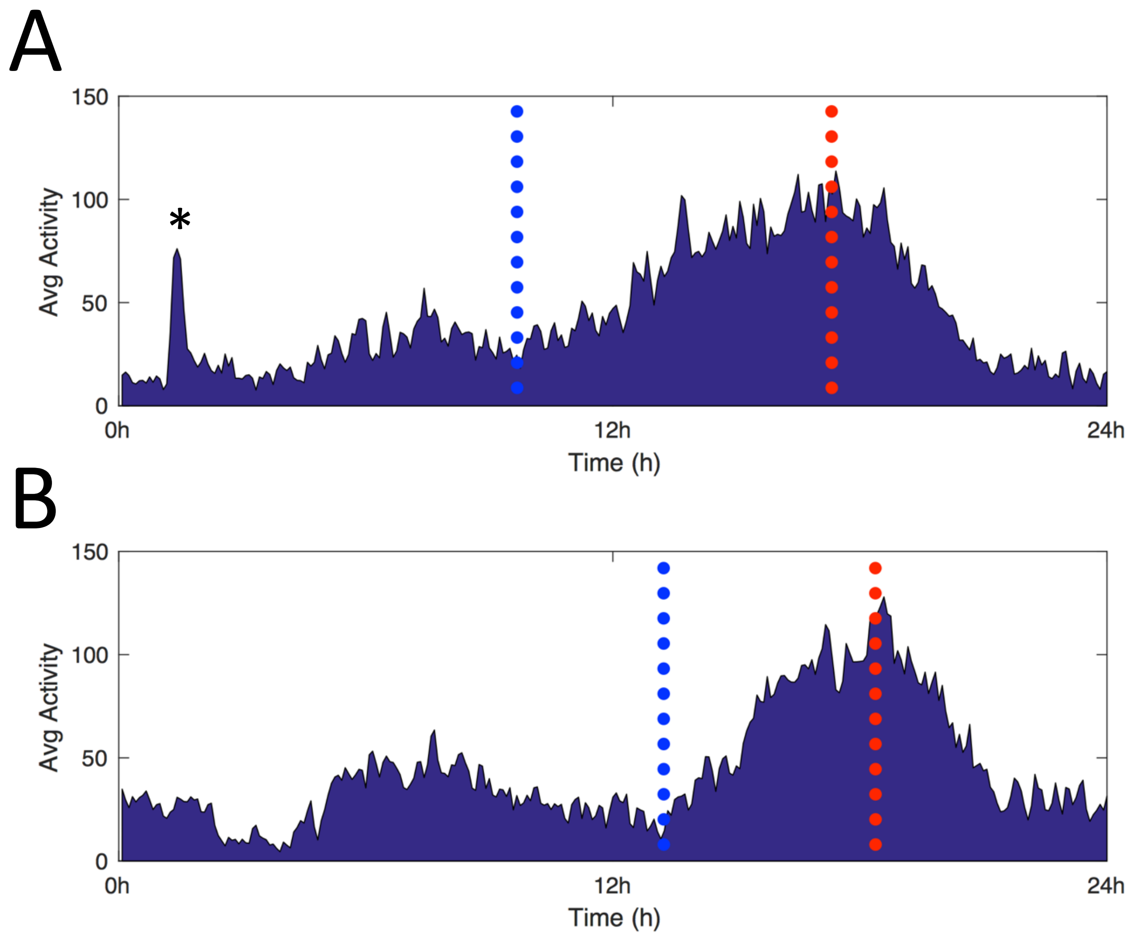

Figure S3. Average Fly Locomotor Activity During FR2
Average locomotor behaviour in wild type flies during the first 2 days of free run following 6h misalignment of LD:TC (A) and 6h LD shift in constant temperature (B). Average actograms covering the entire experiment can be seen in Figure S2 E,F. Red dashed line denotes peak phase; blue dashed line denotes activity onset. Black asterisk denotes startle behaviour elicited by onset of temperature at the beginning of free run following conflicting conditions.

Figure S4 (Related to Figure 4)

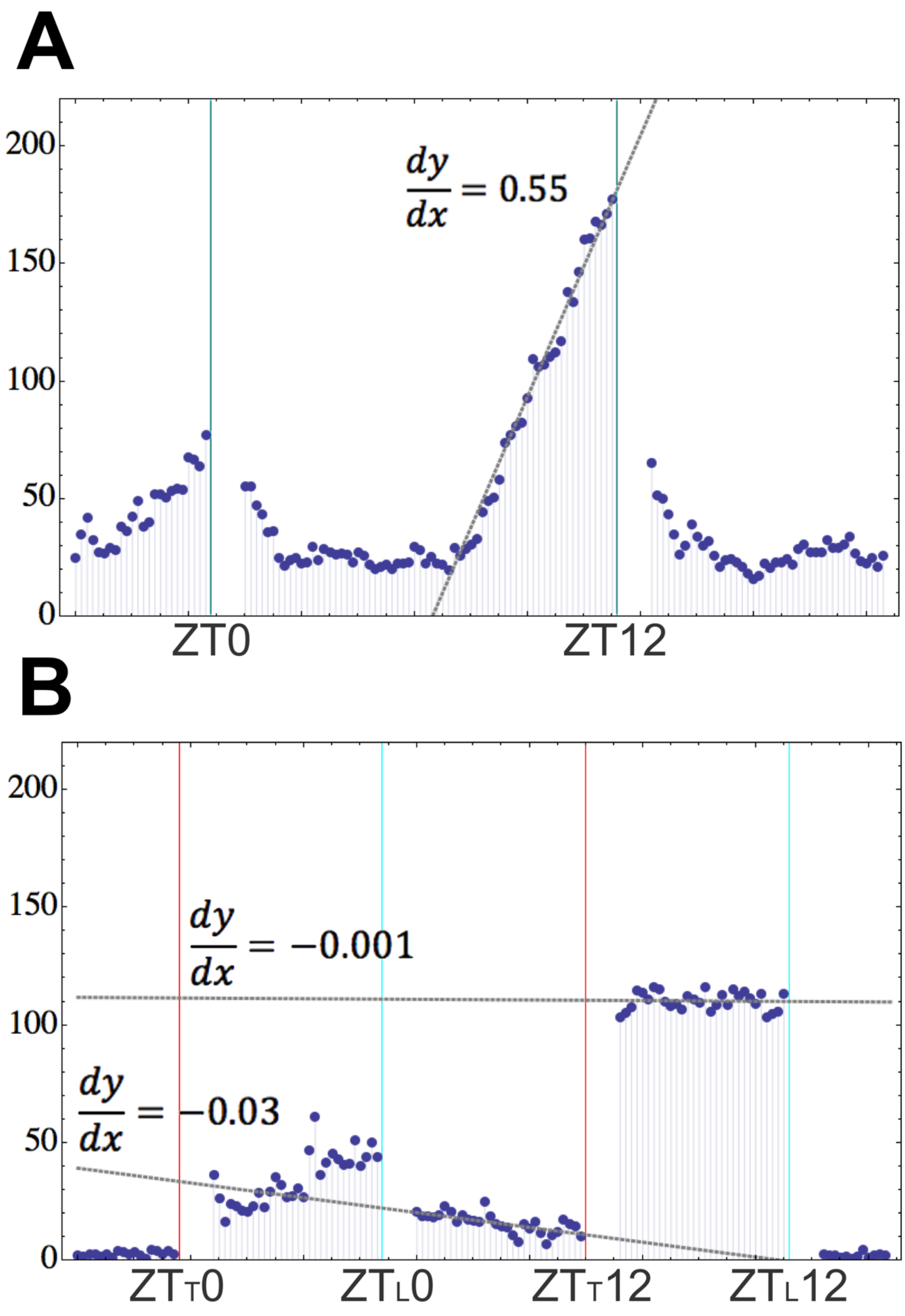

Figure S4. Evening Activity Gradient Quantification in wild type Flies
Linear regression is applied to the period of activity prior to Zeitgeber offset using MLE. Data points at, and 1hr after, the Zeitgeber transitions are excluded from the analysis. (A) When light and temperature are in-phase, $ZT_T = ZT_L$, and the linear increase of activity towards Zeitgeber offset is comparatively steep, showing typical locomotor entrainment. (B) When light and temperature are out-of-phase by 6hr, $ZT_T \neq ZT_L$, and thus we fit two linear regressions: one for the activity preceding temperature offset (red line), and one for the activity preceding light offset (cyan line).

Table S1 (Related to Figure 2). Statistics to accompany antibody staining data presented in Fig. 2.

One-Way ANOVA: P-values

Part I	s-LN_v	l-LN_v	LN_d	DN_3	DN_1	DN_2
Canton S	7.2×10^{-13}	6.6×10^{-27}	3.4×10^{-32}	5.8×10^{-37}	2.2×10^{-44}	2.9×10^{-21}
w;cry^{02}	2.1×10^{-13}	2.2×10^{-13}	1.7×10^{-22}	3.7×10^{-14}	6.9×10^{-29}	5.1×10^{-21}
**Part III**	**s-LN_v**	**l-LN_v**	**LN_d**	**DN_3**	**DN_1**	**DN_2**
Canton S	3.3×10^{-17}	5.0×10^{-16}	5.9×10^{-14}	1.5×10^{-13}	5.2×10^{-18}	3.4×10^{-7}
w;cry^{02}	3.8×10^{-17}	3.7×10^{-11}	3.4×10^{-19}	9.9×10^{-30}	5.9×10^{-21}	3.7×10^{-16}

Two-tailed T-Test Between ZT_T21 (ZT_L15) and ZT_T3 (ZT_L21) in Sensory Conflict: P-values

	s-LN_v	l-LN_v	LN_d	DN_3	DN_1	DN_2
CantonS	1.4×10^{-14}	1.2×10^{-9}	5.8×10^{-12}	5.0×10^{-5}	1.6×10^{-3}	1.4×10^{-4}
w;cry^{02}	1.4×10^{-3}	1.8×10^{-4}	5.0×10^{-7}	3.0×10^{-16}	1.4×10^{-5}	1.5×10^{-6}

N numbers (brain hemispheres)

	Part I	s-LN_v	l-LN_v	LN_d	DN_3	DN_1	DN_2
ZT_T21	Canton S	19	19	20	21	21	21
	w;cry^{02}	11	11	12	11	12	12
ZT_T3	Canton S	25	23	26	24	25	21
	w;cry^{02}	18	18	18	18	18	16
ZT_T9	Canton S	18	18	18	18	18	13
	w;cry^{02}	23	23	23	24	24	24
ZT_T15	Canton S	21	22	23	24	23	21
	w;cry^{02}	12	12	12	12	12	12
	**Part III**	**s-LN_v**	**l-LN_v**	**LN_d**	**DN_3**	**DN_1**	**DN_2**
ZT_T21	Canton S	33	34	34	34	32	31
	w;cry^{02}	19	20	20	14	14	16
ZT_T3	Canton S	33	32	35	35	34	26
	w;cry^{02}	25	26	26	25	24	22
ZT_T9	Canton S	18	18	17	19	20	09
	w;cry^{02}	23	24	24	24	24	24
ZT_T15	Canton S	28	28	32	32	32	32
	w;cry^{02}	16	16	16	17	16	16

Table S2. (Related to Figure 3) Quantification of free running activity rhythms in Part IV of the Experimental Regime

	τ(h)	RS	% Rhythmic	n
6h Conflict	24.6 ± 0.10	6.1 ± 0.2	100	38
LD Shift	24.3 ± 0.12	5.7 ± 0.19	95.7	44

Free running period values their significance (RS values) were determined as described in Supplemental Experimental Procedures. Period values associated with RS values ≥ 1.5 were considered rhythmic (Levine et al 2002)

Supplemental Experimental Procedures

Fly Strains

Canton S flies were used as wild type flies. Cryptochrome mutants were *w;cry*^{02} and *w;cry*^{01}/*cry*^{b} (Dolezelova et al., 2007; Stanewsky et al., 1998). For rescue experiments, *Clk856-gal4* (Gummadova et al., 2009), and *tim-gal4:27* (Kaneko and Hall, 2000), were crossed into a homozygous mutant *cry*^{b} background (Stanewsky et al., 1998) using appropriate balancer chromosomes and dominant markers. These *gal4* driver lines were then crossed to homozygous *cry*^{01} flies carrying *UAS-cry24.5* on chromosome *2* (Emery et al., 1998). F1 *Clk856-gal4/* or *tim-gal4:27/UAS-cry24.5, cry*^{b}/*cry*^{01} males were analyzed behaviorally as described below. Flies were reared under LD 12:12 cycles on *Drosophila* medium (0.8% agar, 2.2% sugar-beet syrup, 8.0% malt extract, 1.8% yeast, 1.0% soy flour, 8.0% corn flour, and 0.3% hydroxybenzoic acid) at 25°C and 60% humidity. Only male flies at an age of 3 to 6 days were used in experiments.

Activity Monitoring

Flies were individually placed into small glass recording tubes containing 5% sucrose and 2% agar medium, which occupied approximately one third of the tube. These tubes were then loaded into MB5 activity monitors (Trikinetics, Waltham, USA), with nine infrared beam detectors separated by 3mm directed at each activity tube. An interruption of the infrared light beam by the movement of a fly produced a signal, which was then recorded by a microprocessor. The number of beam breaks was recorded for each fly in 5-minute time bins and summed into bin counts. Thus, 12 activity scans were obtained for each fly per hour.

Monitors were placed in light- and temperature-controllable incubators (Percival) for the duration of the experiments. 12:12 LD was generated through square wave transition

between ~2500 and 0 lux respectively. 12:12 TC was achieved through transitions between 26°C (ON) and 16°C (OFF) occurring over ~10min. Environmental conditions were recorded with an environmental monitor placed inside the incubator. These were checked to validate scheduled conditions. (Details of specific experimental designs described at relevant points in Results).

Data Analysis

Activity of individual flies and average activity of the population were plotted as double actograms using the Matlab Flytoolbox library. Period length and proportion of rhythmic animals during free-running conditions, were calculated using autocorrelation in the Matlab Flytoolbox library (Levine et al., 2002). The autocorrelation output 'Rhythms Strength' (RS) serves as an estimate of the rhythm strength associated with each period value. In this study flies with RS values ≥ 1.5 were considered to be rhythmic (Levine et al., 2002, Table S2). To determine and compare the phase of the activity peaks during the two free-run parts of the experiment, circular statistics and phase plots using the same Matlab Flytoolbox library were used (Levine et al., 2002). All other analysis was carried out in Wolfram Mathematica using bespoke programs written for the purposes of this study (details of which are described in relevant sections of this report).

Quantification of Entrained Behavior

Analysis of locomotor behavior under entrained conditions is inherently challenging as observed behavior must be a result of both circadian drive and direct sensory effects (e.g. startle behavior and masking). It is common within the field to assess the anticipatory behavior prior to Zeitgeber offset - the so-called 'evening activity'. A simple measure of this evening behavior is a linear increase of activity prior to Zeitgeber offset.With this in mind, maximum likelihood estimation was used to best fit the linear regression, $y = a + bx$, to the

activity bout immediately prior to both light and temperature evening (Figure S3). The regression analysis was applied only to data points that displayed linearity preceding offset of the Zeitgeber in question. In cases where Zeitgeber offset for light and temperature were in close proximity, care was taken not to include evening activity for one stimulus in the analysis for the second. Thus, during very small or very large conflicts, fewer data points were available for fitting the later Zeitgeber, which translated into larger confidence intervals for these time points.

Immunostaining and Quantification

Flies collected at four time points during the in-phase and out-of-phase conditions (corresponding to ZT3, ZT9, ZT15 and ZT21 of the in-phase condition) were fixed in 4% paraformaldehyde in 0.1M phosphate buffer (PB) with 0.1% Triton X-100 (PBS-T) for 2.5h at room temperature. Flies were then rinsed three times in PB, and the brains subsequently dissected in PB. Brains were then blocked in 5% normal goat serum (NGS) in 0.5% PBS-T at 4˚C for 36h before incubation in primary antibodies for 48h at 4˚C. Double staining was conducted with primary antibodies: rabbit anti-PER (1:1500) (Stanewsky et al., 1997), and mouse anti-PDF (1:500) (DSHB). Secondary fluorescence-conjugated antibodies were alexaFluor 488 and alexaFluor 647 (purchased from Thermo Fisher Scientific), and were both diluted 1:300 in 0.5% PBS-T. Secondary antibodies were applied after washing six times in 0.5% PBS-T. After incubation with secondary antibodies, the brains were washed six times in 0.5% PBS-T and mounted in Vectashield (Vector Labs) mounting medium. The fluorescence signals of the whole mount brains were visualized using a Leica SP8 laser scanning confocal microscope.

Staining was quantified as described previously (Rieger et al., 2006), and a final staining index was calculated for each cell group:

$$SI = (Group\ mean\ pixel\ intensity - background) \times \frac{number\ of\ stained\ cells}{maximum\ number\ of\ cells\ in\ group}$$

The maximum number of cells for the different neuronal groups was as follows:

s-LN_v, 4; l-LN_v, 5; LN_d 7; DN_1, 17; DN_2, 2. Owing to the large number of DN_3 neurons, SI for this subgroup was calculated as $group\ mean\ pixel\ intensity - background$.

Statistical Methods

All statistical methods are described at relevant points in the text and supplemental information. In brief, phase comparisons of free-running activity rhythms were conducted using circular phase statistics using the Matlab Flytoolbox library (Levine et al., 2002). Here, activity data is smoothed using a low-pass filter, and average peak phase across two consecutive days is calculated for each fly. The results are then plotted in polar coordinates, and a dispersion test used to determine whether the two distributions (FR1 and FR2) differ significantly in angular deviation from their respective means.

For analysis of immunostaining data, one-way ANOVA was used to examine an effect of ZT on PER staining intensity. In addition to this, two-tailed t-test was used to compare staining of neuronal subgroups between time points as shown in Figure 2 and Table S1.

Supplemental References

Dolezelova, E., Dolezel, D., Hall, J.C., 2007. Rhythm Defects Caused by Newly Engineered Null Mutations in Drosophila's cryptochrome Gene. Genetics 177, 329–345. doi:10.1534/genetics.107.076513

Emery, P., So, W. V, Kaneko, M., Hall, J.C., Rosbash, M., 1998. CRY, a Drosophila clock and light-regulated cryptochrome, is a major contributor to circadian rhythm resetting and photosensitivity. Cell 95, 669–679. doi:10.1016/S0092-8674(00)81637-2

Gummadova, J.O., Coutts, G.A., Glossop, N.R.J., 2009. Analysis of the Drosophila Clock promoter reveals heterogeneity in expression between subgroups of central oscillator cells and identifies a novel enhancer region. J. Biol. Rhythms 24, 353–367. doi:10.1177/0748730409343890

Kaneko, M., Hall, J.C., 2000. Neuroanatomy of cells expressing clock genes in Drosophila: Transgenic manipulation of the period and timeless genes to mark the perikarya of circadian pacemaker neurons and their projections. J. Comp. Neurol. 422, 66–94. doi:10.1002/(SICI)1096-9861(20000619)422:1<66::AID-CNE5>3.0.CO;2-2

Levine, J., Funes, P., Dowse, H., Hall, J., 2002. Signal analysis of behavioral and molecular cycles. BMC Neurosci. 3, 1. doi:10.1186/1471-2202-3-1

Rieger, D., Shafer, O.T., Tomioka, K., Helfrich-Förster, C., 2006. Functional analysis of circadian pacemaker neurons in Drosophila melanogaster. J. Neurosci. 26, 2531–43. doi:10.1523/JNEUROSCI.1234-05.2006

Stanewsky, R., Frisch, B., Brandes, C., Hamblen-Coyle, M.J., Rosbash, M., Hall, J.C., 1997. Temporal and spatial expression patterns of transgenes containing increasing amounts of the Drosophila clock gene period and a lacZ reporter: mapping elements of the PER protein involved in circadian cycling. J. Neurosci. 17, 676–696.

Stanewsky, R., Kaneko, M., Emery, P., 1998. The cryb Mutation Identifies Cryptochrome as a Circadian Photoreceptor in Drosophila. Cell 95, 681–692.
