# Supplementary material for: Sensory Conflict Disrupts Activity of the Drosophila Circadian Network
Source: Cell Rep. 2016 Nov 8;17(7):1711–8. doi: 10.1016/j.celrep.2016.10.029 (PMC5120367; doi:10.1016/j.celrep.2016.10.029)
Supplement: Document S2. Article plus Supplemental Information [file mmc2.pdf]

Cell Reports

Report

Sensory Conflict Disrupts Activity of the *Drosophila* Circadian Network

Graphical Abstract

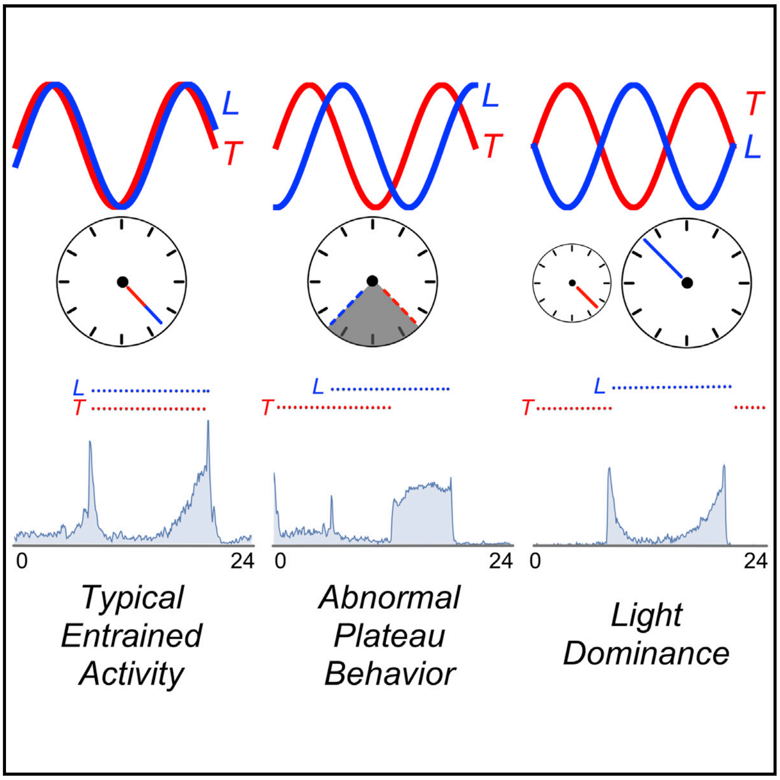

Authors

Ross E.F. Harper, Peter Dayan, Joerg T. Albert, Ralf Stanewsky

Correspondence

joerg.albert@ucl.ac.uk (J.T.A.), r.stanewsky@ucl.ac.uk (R.S.)

In Brief

Circadian clocks allow bodily functions to occur at optimal times of day and need to be synchronized with the local environment. Harper et al. examine how circadian clocks respond to situations of sensory conflict and show that in *Drosophila,* intermediate (5–7 hr) offsets between light and temperature cycles lead to an altered activity profile, underpinned by a drastically compromised molecular clock.

Highlights

- Conflicting light and temperature cycles lead to abnormal, plateau-like locomotor behavior
- Plateau-like behavior is accompanied by a collapse of the molecular circadian clock
- Temperature cues dominate during small light and temperature misalignments
- Light cues dominate during large light and temperature misalignments

Harper et al., 2016, Cell Reports *17*, 1711–1718
November 8, 2016 © 2016 The Author(s).
http://dx.doi.org/10.1016/j.celrep.2016.10.029

Cell Reports
Report

OPEN ACCESS
CellPress

Sensory Conflict Disrupts Activity of the *Drosophila* Circadian Network

Ross E.F. Harper,[1,2] Peter Dayan,[3] Joerg T. Albert,[1,2,4,*] and Ralf Stanewsky[4,5,6,*]
[1]Centre for Mathematics, Physics and Engineering in the Life Sciences and Experimental Biology (CoMPLEX), University College London, London WC1E 6BT, UK
[2]Ear Institute, University College London, London WC1X 8EE, UK
[3]Gatsby Computational Neuroscience Unit, University College London, London W1T 4JG, UK
[4]Department of Cell and Developmental Biology, University College London, London WC1E 6BT, UK
[5]Present address: Institute for Neuro and Behavioral Biology, Westfälische Wilhelms University, 48149 Münster, Germany
[6]Lead Contact
*Correspondence: joerg.albert@ucl.ac.uk (J.T.A.), r.stanewsky@ucl.ac.uk (R.S.)
http://dx.doi.org/10.1016/j.celrep.2016.10.029

SUMMARY

Periodic changes in light and temperature synchronize the *Drosophila* circadian clock, but the question of how the fly brain integrates these two input pathways to set circadian time remains unanswered. We explore multisensory cue combination by testing the resilience of the circadian network to conflicting environmental inputs. We show that misaligned light and temperature cycles can lead to dramatic changes in the daily locomotor activities of wild-type flies during and after exposure to sensory conflict. This altered behavior is associated with a drastic reduction in the amplitude of PERIOD (PER) oscillations in brain clock neurons and desynchronization between light- and temperature-sensitive neuronal subgroups. The behavioral disruption depends heavily on the phase relationship between light and temperature signals. Our results represent a systematic quantification of multisensory integration in the *Drosophila* circadian system and lend further support to the view of the clock as a network of coupled oscillatory subunits.

INTRODUCTION

Circadian networks generate endogenous rhythms that optimize the behavior of organisms for a periodic environment. However, environmental fluctuations are themselves intrinsically variable, changing across seasons and latitudes. A reliable circadian pacemaker must therefore possess the capacity to synchronize its oscillations to periodic environments without being disturbed by short and sporadic changes, as exist under natural conditions. In the fruit fly, *Drosophila melanogaster*, the two most potent clock-resetting signals, or Zeitgebers (ZG), are light-dark (LD) and temperature cycles (TCs). Individually and together, these two sensory modalities can entrain locomotor activity rhythms as well as molecular rhythms in clock cell groups throughout the fly (Dubruille and Emery, 2008; Glaser and Stanewsky, 2005; Plautz, 1997; Sehadova et al., 2009; Wheeler et al., 1993; Yoshii et al., 2009; Zerr et al., 1990). This poses a question of sensory integration: how are different, and potentially conflicting, sources of information integrated by the clock to compute circadian time and produce a coherent behavioral output?

Coordinated circadian behavior in *Drosophila* emerges from the concerted activity of a network of ~150 clock neurons located in the central nervous system, which are endowed with the intracellular capacity for circadian rhythmicity (Peschel and Helfrich-Förster, 2011). A traditional view of the clock highlights the small Pigment Dispersing Factor (PDF)-positive lateral ventral neurons (s-LN_vs) as autonomous pacemakers, which impose rhythmicity on a more passive remainder of the network (Renn et al., 1999). The reality, however, is likely to be more complicated. Indeed, experimental conditions heavily influence both the supposed identity of these clock "masters" and the precise network hierarchy reported (Helfrich-Förster et al., 2007).

Laboratory conditions typically treat ZGs in a singular manner; circadian networks, however, operate subject to multisensory challenges. This concept has been embraced by a small number of previous studies, which form the foundation of our work (Currie et al., 2009; Miyasako et al., 2007; Yoshii et al., 2010). In one, LD and TCs were misaligned by 12 hr (Yoshii et al., 2010)—an antiphasic relationship that represents the largest possible disparity between two 24-hr environmental oscillators. During this extreme sensory conflict, activity rhythms of wild-type flies entrain preferentially to the light stimulus, leading to the conclusion that this cue is dominant (a prevailing view in the field). However, in a similar study investigating antiphasic LD:TC, temperature was found to have a more substantial circadian effect, advancing the onset of evening locomotor activity (Currie et al., 2009). Moreover, field studies exploiting naturalistic environmental fluctuations demonstrate a more prominent role of temperature in locomotor entrainment (Vanin et al., 2012). The situation thus remains unclear. The analysis of one single signal disparity is insufficient to fully probe the possible coupling at play in the *Drosophila* circadian system.

In another study, a smaller degree of environmental misalignment was implemented using a 6-hr advance of TC relative to LD (Miyasako et al., 2007). However, the comparatively small

This is an open access article under the CC BY license (http://creativecommons.org/licenses/by/4.0/).

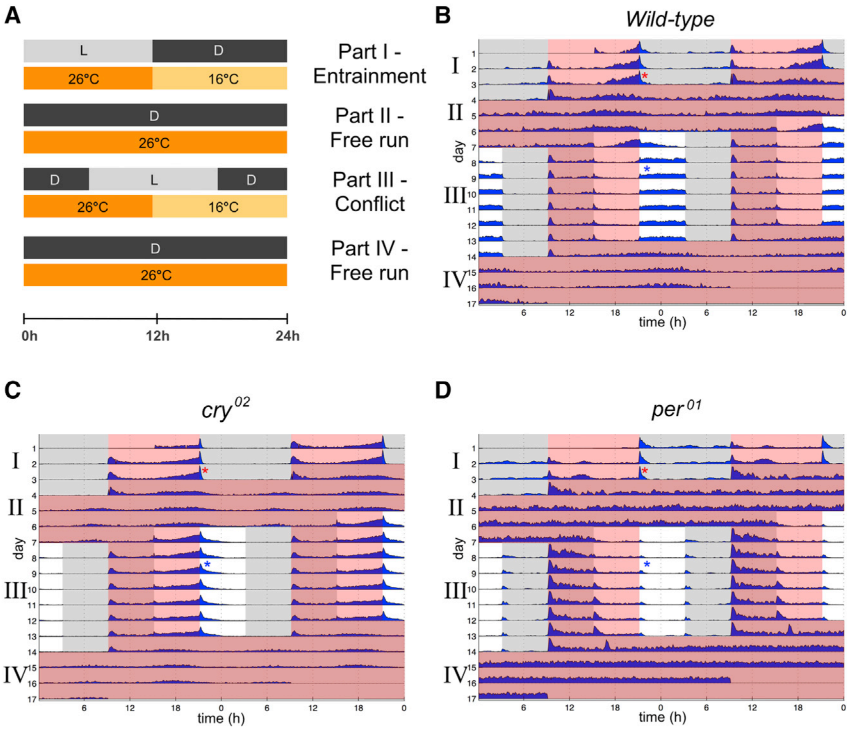

Figure 1. Locomotor Behavior during Sensory Conflict

(A) Experimental regime in which environmental conditions followed 3 days of 12-hr:12-hr LD and TC (16:26°C) in-phase (I), 3 days of free run in DD at 26°C (II), 7 days of out-of-phase 12-hr:12-hr LD and TC (16:26°C) via 6-hr delay of LD (III), followed by 3 days of free run in DD at 26°C (IV).

(B–D) Average actograms of wild-type (B) ($n = 46$), *cry*^{02} (C) ($n = 44$), and *per*^{01} (D) ($n = 32$). Red asterisk denotes representative evening behavior in part I; blue asterisk denotes representative pseudo-evening behavior in part III. Clock-less *per*^{01} flies show only brief startle responses to the sudden environmental changes and otherwise display arrhythmic behavior (C).

See Figure S1 and S2 for individual fly data and genetic controls.

amplitude TC (20:25°C), for what is regarded as the weaker of the two ZGs in *Drosophila* (Yoshii et al., 2010) is likely to have been insufficient to distinguish subtle signal averaging effects from background noise, especially given the much larger temperature ranges found in nature (Vanin et al., 2012). Again, this might explain the relatively undisturbed light-aligned locomotor activity observed under these specific conditions.

To better understand the effect of environmental phase relationships on circadian clock function, we assessed circadian locomotor behavior during misaligned LD and TC using finer gradations of sensory conflict and greater diurnal fluctuations in both cues. Furthermore, we compared wild-type flies to *cry*-null mutants, removing the key contribution made by the circadian photoreceptor Cryptochrome (CRY) to light entrainment of the clock (Stanewsky et al., 1998). We hypothesized that any effect of multisensory integration would be markedly diminished in *cry* mutants, owing to a reduced weight of the light-dependent input pathway and relative enhancement of the temperature cue (Gentile et al., 2013).

RESULTS

Sensory Conflict Disrupts Normal Daily Locomotor Activity

While recent studies have aimed to generate more naturalistic environmental transitions (e.g., Vanin et al., 2012), our study of the mechanistic bases of ZG integration requires the establishment of deliberately *unnatural* experimental conditions. Note that we refer to cue misalignment as the absolute distance, in hours (*delta* time, or Δt), between onset/offset of two cyclic 12-hr:12-hr signals. For example, $\Delta t_{L,T}$ = 3 hr denotes that light onset/offset occurs 3 hr after temperature.

Wild-type flies (Canton S) and *cry*-null mutants (*cry*^{02}) were subjected to an environmental regime comprising aligned LD:TC (part I, $\Delta t_{L,T}$ = 0 hr), followed by a 6-hr delay of LD with respect to TC (part III, $\Delta t_{L,T}$ = 6 hr), interspersed or followed by free running conditions to assess stability of endogenous rhythms (part II and part IV, outlined in Figure 1A). As is standard practice for observing endogenous activity rhythms, free running conditions comprised constant darkness and constant warmth (26°C—*Drosophila*'s preferred ambient temperature [Sayeed and Benzer, 1996]) to mitigate any negative masking effect of cold temperatures on overall activity levels.

In part I, locomotor behavior in wild-type and *cry*^{02} flies both displayed a characteristic bimodal profile, showing an evening peak of activity that coincided with the end of photo/thermophase (Figures 1B and 1C). These entrained rhythms persisted in free-running conditions (part II). In part III, a 6-hr misalignment between LD and TC was introduced via a 6-hr delay of LD relative to part I (leaving TC unchanged). Under sensory conflict, circadian locomotor behavior in wild-type flies was drastically altered, exhibiting a plateau of sustained activity between temperature offset and light offset, bordered by periods of inactivity (Figure 1B). The activity pattern continued for the duration of the conflict and was also seen at the level of individual flies, and across multiple repeats (Figures S1 and S2E). A key facet of this activity pattern is the absence of any evening anticipation to either the light or the temperature cue. For ease, we refer to this abnormal locomotor behavior as "plateau" (P) behavior. Importantly, P behavior depends on a functional clock as it cannot be observed in *per*^{01} mutants (Figure 1D). That P behavior is not merely induced by masking is also apparent from comparing the free running behavior in part IV with that in part II (Figures 1B and S2E).

The P behavior observed in wild-type flies was not present in *cry*^{02} mutants during conflict conditions, which instead

displayed the typical ramping increase of activity, peaking at temperature offset (Figure 1C). This suggests these flies predominantly entrained to TC. However, we do note the behavioral profile is slightly altered from that in part I, for instance, including an extended period of activity after temperature offset. The conflicting regime (and therefore the periodic presence of light) appears to have had some effect, albeit greatly reduced, on the behavior of cry^{02} mutants. This observation is consistent with the existence of *cry*-independent light entrainment pathways (Yoshii et al., 2015).

To test whether the absence of P behavior in cry^{02} mutants was indeed due to the absence of CRY, we rescued *cry* expression in all clock cells or all clock neurons (*tim-gal4/* and *Clk856-gal4/UAS-cry;cry*^{b}/*cry*^{01}, respectively). Rescue flies displayed activity rhythms that more closely resembled the wild-type than the cry^{02} pattern—inactive prior to temperature offset, with a bout of activity between temperature and light offset (Figure S2). These data suggest that it is indeed the integration of two potent, yet conflicting input signals to the clock—one photic and the other non-photic—that underlies the abnormal behavioral output observed in sensory conflict.

Sensory Conflict Disrupts Endogenous Oscillations in the Central Clock Network

Cytological staining for clock gene products has revealed the location of the central circadian network in *Drosophila* (Ewer et al., 1992; Frisch et al., 1994; Zerr et al., 1990), which can be further classified into seven distinct cell groups: small and large ventral lateral neurons (s-LN_v, l-LN_v), dorsal lateral neurons (LN_d), the first, second, and third dorsal neuron groups (DN_1, DN_2, and DN_3) and the lateral posterior neurons (LPNs) (Nitabach and Taghert, 2008). While there are likely to be additional subdivisions within the network (Peschel and Helfrich-Förster, 2011), our study of multisensory processing in the fly brain adopted the prevailing, and well-supported, network architecture. Indeed, it has been shown previously that the molecular rhythms of clock neurons expressing CRY appear to entrain preferentially to light, whereas the CRY-negative DN_2 and LPN subgroups entrain preferentially to temperature in 12-hr conflicting LD:TC (Yoshii et al., 2010).

To examine the molecular and neuronal substrates of the pronounced P behavior, we carried out antibody staining for the clock protein PERIOD (PER) in the *Drosophila* brain during 6-hr misaligned LD:TC. PER immunostaining of clock neurons was performed at four time points evenly spaced across 24 hr (ZT3, ZT9, ZT15, and ZT21) for both part I and part III of the experimental regime. In flies that have entrained to a given ZG, maximum and minimum staining intensity is expected at ZT21 and ZT9 respectively (Yoshii et al., 2009) (note that during sensory conflict ZT_L and ZT_T refer to ZT specified by light and temperature, respectively).

During in-phase LD:TC (part I), wild-type flies showed the expected strong PER oscillations in all neuronal subgroups with a peak at ZT21 and a trough at ZT9 (Figures 2A, left, and 2C, top). In cry^{02} mutants, PER cycled with the same phase, but with lower amplitude (Figure 2B, left), consistent with previous findings that light and temperature synergistically entrain molecular rhythms (Yoshii et al., 2009).

By contrast, during conflict, we observed a striking collapse in the amplitude of PER oscillations for all neuronal subgroups in wild-type flies (Figures 2A, right, 2C, bottom, and 2D). Furthermore, inspecting the residual low-amplitude PER oscillations, there appeared to be a clear shift in the peak of the s-LN_v, l-LN_v, LN_d, and DN_1 to ZT_L21, suggesting at least partial entrainment of these neurons to LD. In contrast, the CRY-negative DN_2 and DN_3 remained phase-locked to TC, displaying peak PER expression at ZT_T21. We did not notice any obvious phase heterogeneity within each neuronal subgroup (see, for example, the DN_2 and LN_d in Figure 4C). In cry^{02} mutants under conflict conditions, molecular rhythms remained comparable to part I (Figures 2B, right, 2D). This echoes our behavioral findings, suggesting that the altered molecular rhythms observed in wild-type flies result from the integration of conflicting inputs to the clock network, and that such conflicts can be avoided by weakening one of the input pathways, as in cry^{02} mutants.

Sustained Effects of Sensory Conflict on the Circadian Clock

Considering the drastic effects of sensory conflict on behavior and molecular clock oscillations, one would expect alterations to the underlying state of the circadian clock. This should manifest itself during constant conditions. We therefore analyzed the consequences of sensory conflict (part III) on the final free run section (part IV). We compared overall rhythmicity and peak phase during free run, with that of control flies that had not experienced sensory conflict. These control flies were initially exposed to the identical in-phase LD:TC and free-running conditions (part I and part II), before being subjected to a 6-hr delayed LD cycle at constant 26°C (part III, $\Delta t_L = 6$ hr) and subsequent release into the final free run (part IV, constant darkness [DD] at 26°C) (Figure S2F). While we did not observe any effects on overall rhythmicity or period length (Table S2), we did notice an advance of the activity peak in flies experiencing sensory conflict compared to those that were shifted with light at constant temperature (Figures 3A, S2E, S2F, and S3). To quantify this apparent effect of the (un-shifted) temperature cue, we determined the magnitude of the phase difference between activity rhythms in free run part II and part IV for sensory conflict and control flies using circular phase analysis (Levine et al., 2002a, Experimental Procedures). As expected, both groups displayed almost identically phased activity peaks during part II (2.4 and 2.2 hr before light and temperature onset in part I, respectively). In contrast, in the free-run (part IV) following sensory conflict, peak activity was delayed by 5.3 hr, while the peak of control flies was delayed by 7.1 hr (Figure 3). Thus, exposure to conflicting ZGs diminished the degree of activity phase shift by almost 2 hr. This observation is consistent with theoretical considerations of the clock as coupled oscillatory subunits, which predict that the resulting equilibrium phase following conflicting input is some weighted average of the two inputs. This would act to reduce the degree of phase shift compared to synchronization with the 6-hr delayed LD alone.

Robustness of the Clock Network to Conflicting Inputs

A recent study by Yao and Shafer (2014) suggests that the *Drosophila* central clock network is resilient to period

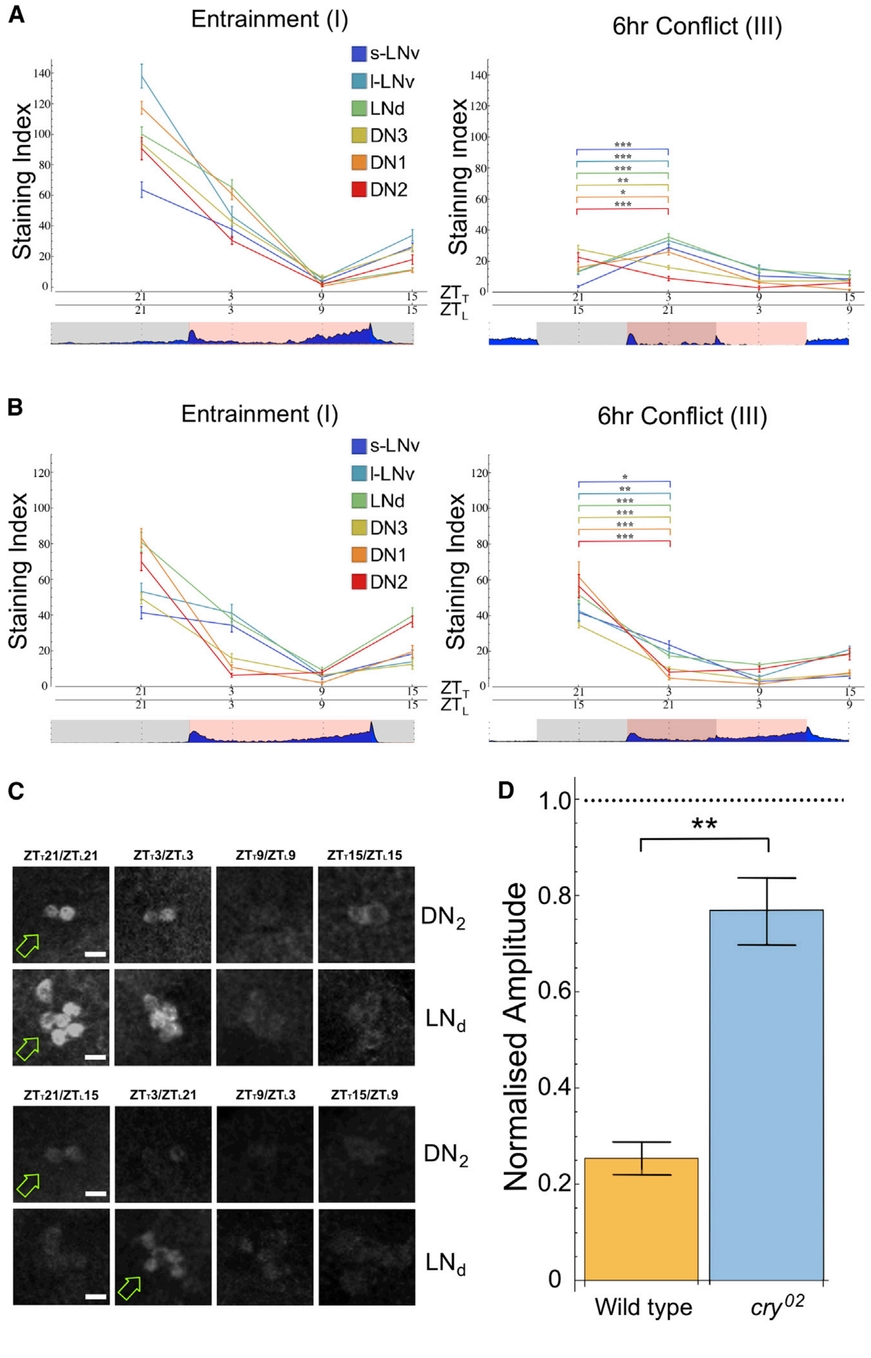

Figure 2. Central Clock Molecular Rhythms during Sensory Conflict

(A and B) PER immunostaining of wild-type (A) and cry^{02} (B) brains during entrainment (left: TC and LD in sync) and 6-hr conflict (Right) conditions. One-way ANOVA reveals a significant effect of ZT on PER staining intensity under in-phase and out-of-phase conditions in both genotypes ($p < 1 \times 10^{-7}$ in all clock neuronal groups). During 6-hr conflict, t test reveals significant differences between the first two time points plotted for all neuronal subgroups in wild-type and cry^{02}. Dissociation in peak staining between different neuronal groups occurred in wild-type, but not in cry^{02} (see also Table S1).

(C) PER staining in the DN_2 and LN_d cell groups in wild-type brains during entrainment (top) and 6-hr conflict (bottom) conditions. Scale bar, 5 μm. Green arrows mark maximum staining for each cell group.

(D) Average amplitude of neuronal subgroup oscillations during sensory conflict (part III) divided by that during entrainment conditions (part I) in wild-type and cry^{02}. A score of 1 denotes no change between conditions.

All error bars represent SEM ($p < 0.01$*$p < 0.001$**$p < 0.0001$***).

discrepancies between neuronal subgroups, such as PDF-negative and PDF-positive neurons. Indeed, it was shown that coherent activity rhythms could still be generated, provided the period length mismatch between the cell groups was less than ~2.5 hr. Having shown that 6-hr-misaligned LD:TC generates P activity patterns (Figure 1B) associated with a severe collapse of PER oscillations in all clock cell groups, and phase differences between light- and temperature-sensitive clock subgroups (Figures 2A and 2D), we went on to explore the consequences of other degrees of sensory conflict for circadian locomotor behavior. Adapting part III of the experimental regime, we conducted a systematic behavioral analysis investigating the effect of varying the magnitude of the LD delay.

When LD:TC misalignment was less than 4 hr, wild-type flies displayed anticipatory behavior and peak activity at the end of thermo-phase, thus appearing to primarily follow the temperature signal (Figure 4A). However, activity persisted after temperature offset into the lights-on phase, suggesting some effect of light on circadian locomotor behavior (reminiscent of that observed in cry^{02} mutants during 6-hr conflict). Fully fledged P behavior emerged at 5- to 7-hr misalignments, with an absence of conventional entrainment to either signal. As the disparity between LD:TC exceeded 7-hr misalignment, P behavior gradually decayed, with a discernible peak of activity observed at light offset during 10-hr misaligned conditions. These results go some way toward explaining previous observations made in antiphasic (i.e., $\Delta t_{L,T}$ = 12 hr) light and temperature (Yoshii et al., 2010)—only during very large sensory conflicts is light the dominant ZG.

We quantified these observations by assessing the gradients of the gradual increase in locomotor behavior that arises toward the offset of ZGs during entrainment. When LD and TC are synchronized, the gradient is positive, consistent with evening anticipation. In our misaligned conditions, the two separate gradients associated with light and temperature offset can be used to gauge the disruption caused (see Experimental Procedures

OPEN ACCESS
CellPress

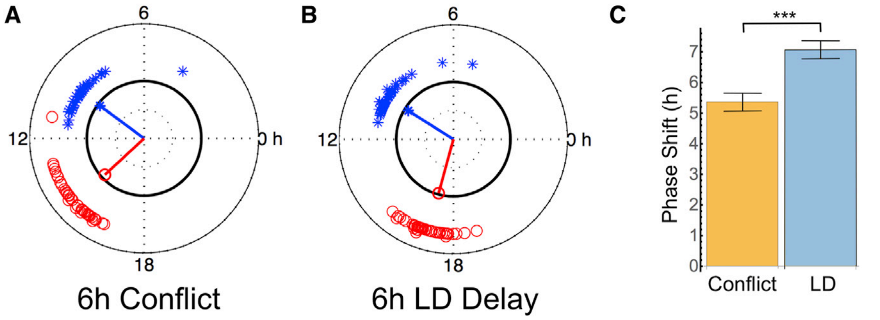

Figure 3. Sustained Effects of Sensory Conflict on Circadian Clock Phase

Comparison of the activity peaks during the free-running parts of the experiment (parts II and IV) preceding and following exposure to (A) 6-hr delayed sensory conflict ($n = 38$, *phase difference* = 5.3 hr, $p < 0.001$) or (B) 6-hr delayed LD cycle at constant 26°C ($n = 46$, *phase difference* = 7.1 hr, $p < 0.001$). Crosses show mean phase of each fly across the first 2 days of free run. Blue shows part II; red shows part IV. Circular statistics as used in Levine et al. (2002a). (C) Bar chart showing magnitude of phase shift between part II and IV in experimental groups (A) and (B). Error bars show SD. $p < 1 \times 10^{-7}$.

and Figure S4). The progressive change in these gradients for temperature and light with misalignment is evident in Figure 4B. P behavior occurs when both gradients approach zero. Entrainment to TC for smaller misalignments, and to LD for the largest misalignments, is also evident from the plot.

In contrast to wild-type flies, the activity rhythms of cry^{02} mutants remain largely entrained to TC, independent of the magnitude of the sensory conflict (Figure 4C). This unwavering temperature preference is again illustrated numerically by the fact that temperature evening gradients remain more positive than light evening gradients for all LD:TC misalignments (Figure 4D).

DISCUSSION

Circadian research in *Drosophila melanogaster* has traditionally treated light and temperature separately. However, clock networks evolved to orchestrate behavior within multisensory environments. Recent studies suggest the existence of multiple independent oscillatory subunits within the fly central clock, each capable of driving activity patterns (Yao and Shafer, 2014). Such distributed architectures tend to exhibit cooperation and/or competition. We here present a systematic and quantitative exploration of the behavioral and molecular effects of conflicting (light/temperature) entrainment regimes on the circadian system. Our paradigm offers a novel route to decompose the circadian network and our findings demonstrate that sensory conflict can—under specific conditions—cause dramatic disruptions to clock output, which have not been reported before.

Although light does indeed dominate temperature for maximal misalignments, smaller delays of LD relative to TC lead to evening activity rhythms in wild-type flies that are predominantly entrained to the temperature cue. These observations are in line with previous reports of temperature also being the critical parameter for morning activity onset in natural conditions (Vanin et al., 2012). Our findings indicate a higher biological relevance for temperature effects on daily behavioral rhythms than previously appreciated. Furthermore, with larger delays of 5–7 hr, typical evening peaks of activity broke down giving way to an abnormal locomotor pattern, which we here refer to as plateau (P) behavior. This P behavior is associated with a drastic reduction in the amplitude of molecular rhythms, as well as dissociation between clock neuronal groups. Importantly, 6-hr sensory conflict also reduced the degree of phase shift compared to that induced by 6-hr delay of light alone, demonstrating that sensory conflict alters the state of the circadian oscillator (Figure 3). It was only during even larger misalignments of 8- to 10-hr that we saw a restoration of more typical evening activity peaks and a reversal of cue preference back to the light signal (cf. Yoshii et al., 2010). Together, these results emphasize the context-dependent nature of ZG dominance. The *Drosophila* circadian system, it appears, is able to generate "wild-type-like" behavioral rhythms only for a limited range of light-temperature phase relationships, i.e., either very small or very large misalignments; intermediate conflicts, however, are not easily accommodated by the clock network.

Throughout our investigation, we have maintained a phase-agnostic approach to our experimental interpretations. It remains unclear how the phase of environmental oscillatory signals translate to circadian phase extracted by the clock. Indeed, temperature typically lags behind light under natural conditions (Boothroyd et al., 2007; Vanin et al., 2012), suggesting that $\Delta t_{L,T} = 0$ hr might not necessarily represent "in-phase" signals as far as the clock is concerned. Pending deeper understanding, we must only treat phase relationships between light and temperature in a relativistic manner. Thus, the coincidence of photo- and thermo-phases should be thought of as an arbitrary reference point (admittedly, one that has been used frequently in the field).

From a mechanistic viewpoint, our molecular data reveal a striking effect of sensory conflict, as 6-hr LD:TC misalignments lead to a drastic reduction in the amplitude of molecular rhythms in all clock neurons. The phase of the remaining low-amplitude oscillations appears largely consistent with that reported previously (Yoshii et al., 2010), revealing a temperature preference of the *cry*-negative DN_2 in wild-type flies. Curiously, residual PER rhythms in the DN_3 also align with TC during sensory conflict. This finding, which might be linked to our particular experimental conditions, has not been reported previously—in 12-hr conflict conditions, PER rhythms in DN_3 preferentially entrain to light (Yoshii et al., 2010). Our results do, however, resemble TIM cycling reported previously during sensory conflict (Miyasako et al., 2007), suggesting a temperature-sensitive property of the DN_3.

At its core, the clock network must perform multisensory integration (MSI). Bayesian methods offer a powerful way to analyze MSI, and, in the context of our results, bring to the fore two key considerations: the relative strengths of different signals; and the

OPEN
ACCESS
CellPress

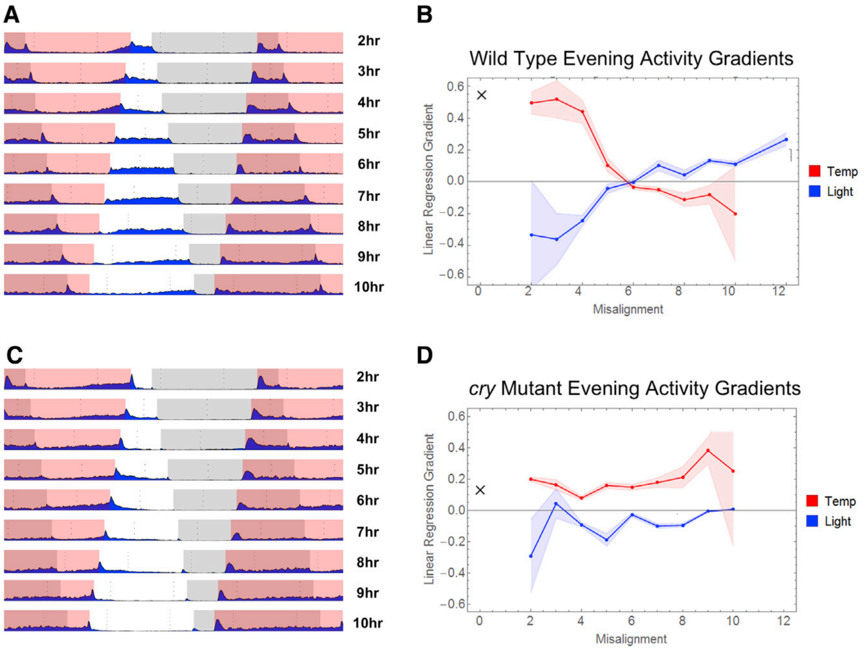

Figure 4. Behavioral Responses to Different Light and Temperature Phase Relationships

Varying degrees of LD:TC misalignments in wild-type (A and B) and cry^{02} flies (C and D) ($45 \leq n \leq 65$). (A and C) Representative days of locomotor behavior taken from average actograms during conflict conditions after activity rhythms had stabilized (part III, days 5–6). (C and D) Gradients of linear regression fit to the period of activity preceding light and temperature cutoffs. Shaded regions denote 95% confidence intervals. Black cross indicates gradient of evening activity during corresponding in-phase condition (see also Figure S4).

possibility that the signals might have different, as opposed to the same, underlying causes.

In the Bayesian characterization of timekeeping, there is a hidden or latent variable (here, the true time of day) whose values are associated with possibly noisy observations (fluctuating light and temperature signals). Different sources of an observation are integrated with different weights of influence according to their respective reliabilities. Weak periodic fluctuations in a source cue provide little reliable evidence about the time of day and so exert little effect over the estimate. This might explain why Miyasako et al. (2007) did not observe P behavior using small fluctuations in the temperature cue during conflict with LD cycles. It would be interesting to investigate whether flies are able *to learn* the reliability of different sources of input and adjust their relative weights accordingly.

Bayesian treatments of MSI also acknowledge the possibility that highly discrepant signals are unlikely to come from the same underlying value of the latent variable (Körding et al., 2007). Depending on the circumstance, inference could then reject one of the signals as being just noise; or it could infer that there is more than one underlying latent variable. In these cases, the smaller the disparity between the signals, the readier inference will be to integrate them. This could explain why aberrant P behavior only arose at conflicts of ~5–7 hr—sufficiently large to disrupt integration, but too small to lead to segregation.

In the case of segregation, the two possibilities have different implications. Rejecting sources as being noise is a choice that itself involves assessments of relative reliabilities, and prior biases. For equally strong sources, prior bias would dominate—which might perhaps favor light. This would be consistent with the observation that the 12-hr LD:TC misalignment used by Yoshii et al. (2010) led to dominance of light entrainment, without substantial behavioral disruption. The second possibility in our case is that two different times of day are inferred, one each associated with light and temperature. This might explain our observed dissociation of distinct populations of clock neurons. Indeed, there may be physiological activities required to occur at certain temperatures, even if at what might be inappropriate light-defined times. This separation could further extend to the peripheral clock network, in which the temperature cue has been shown to have a prominent role in entrainment (Sehadova et al., 2009). It would certainly be intriguing to explore the response of these peripheral clocks to sensory conflict.

Conclusion

Robustness toward a range of variable, and potentially conflicting, inputs is a beneficial property for any sensory network. We show that phase discrepancies between clock neurons can result from sensory conflict, and that in these conditions, the fly clock resists some, but embraces other, misalignments. Network robustness offers obvious advantages in itself, but possible benefits extend beyond this. Resilience might also imply plasticity, allowing different clock cell groups to exhibit autonomy under different conditions, truly optimizing behavior for particular environmental features. Moreover, in nature, the phase relationship between light and temperature might also provide valuable circannual information to the network.

Building on previous studies, we focused on the interplay between light and temperature in *Drosophila.* Our findings, however, are not restricted to these two sensory entrainment pathways, nor are they restricted to the fly. Links between human circadian clock function (and dysfunction) and mental disorders have been made repeatedly, but the directions of the underlying causalities are still unclear (Roenneberg and Merrow, 2016). Most intriguing in this regard is the suggestion that the associations between psychiatric pathologies and the clock partly involve behavioral habits, which alter an individual's exposure to different ZGs (Adan et al., 2012). A more thorough study of multisensory processing in the circadian system, and possible conflicts that can arise therein, therefore stands not only to increase our understanding of the computation of time, but also to enable novel approaches in the treatment, and prevention, of mental disorders.

Other cues, such as mechanical (Simoni et al., 2014) and social (Levine et al., 2002b) ones, have been shown capable of entraining the fruit fly's circadian clock. The case of mechanosensory clock input is particularly interesting as proprioceptive feedback from an individual's own locomotor behavior may in fact contribute back to clock entrainment, blurring the boundaries between network output and input. We look forward to future work further disentangling the complex nature of multisensory processing in biological time-keeping systems.

EXPERIMENTAL PROCEDURES

Activity Monitoring

Locomotor activity rhythms were recorded automatically using the *Drosophila* Activity Monitoring (DAM) system (Trikinetics) as previously described (Glaser and Stanewsky, 2005). See Supplemental Experimental Procedures.

Data Analysis

Raw activity data were scanned using DAM File Scan software and saved into txt files. All analyses were carried out using the MATLAB Flytoolbox library (Levine et al., 2002a) and Wolfram Mathematica. See Supplemental Experimental Procedures.

Immunostaining and Quantification

Flies were collected at four time points during the in-phase and out-of-phase conditions (corresponding to ZT3, ZT9, ZT15, and ZT21 of the in-phase condition), and brains were subsequently incubated with PER antibodies (see Supplemental Experimental Procedures). Quantification of PER signals was conducted without discrimination of sub-cellular localization using ImageJ, as described previously (Rieger et al., 2006). PDF staining served as a useful neuroanatomical marker to distinguish between LN_v and other clock neuronal groups. Statistical tests, including t test and ANOVA, were conducted in Mathematica.

SUPPLEMENTAL INFORMATION

Supplemental Information includes Supplemental Experimental Procedures, four figures, and two tables and can be found with this article online at http://dx.doi.org/10.1016/j.celrep.2016.10.029.

AUTHOR CONTRIBUTIONS

R.E.F.H. conducted the experiments and analysis. R.E.F.H., P.D., J.T.A., and R.S. designed the experiments and authored the paper. J.T.A. is the corresponding author for circadian, computational, and conceptual questions, and R.S. is the corresponding author for circadian, experimental, and molecular issues.

ACKNOWLEDGMENTS

We thank Eva Dolezelova for the cry^{02} mutants and Maite Ogueta-Gutierrez for advice with the dissection and immunohistochemistry experiments. R.E.F.H. received funding from the Engineering and Physical Sciences Research Council (EP/F500351/1). This work was supported by a grant from the European Research Council to J.T.A. (H2020 - ERC-2014-CoG/648709/Clock Mechanics) a BBSRC grant BB/J018589/2, given to R.S., and a grant from the Gatsby Charitable Foundation to P.D.

Received: June 10, 2016
Revised: September 16, 2016
Accepted: October 11, 2016
Published: November 8, 2016

REFERENCES

Adan, A., Archer, S.N., Hidalgo, M.P., Di Milia, L., Natale, V., and Randler, C. (2012). Circadian typology: A comprehensive review. Chronobiol. Int. *29*, 1153–1175.

Boothroyd, C.E., Wijnen, H., Naef, F., Saez, L., and Young, M.W. (2007). Integration of light and temperature in the regulation of circadian gene expression in Drosophila. PLoS Genet *3*, e54.

Currie, J., Goda, T., and Wijnen, H. (2009). Selective entrainment of the Drosophila circadian clock to daily gradients in environmental temperature. BMC Biol. *7*, 49.

Dubruille, R., and Emery, P. (2008). A plastic clock: How circadian rhythms respond to environmental cues in Drosophila. Mol. Neurobiol. *38*, 129–145.

Ewer, J., Frisch, B., Hamblen-Coyle, M.J., Rosbash, M., and Hall, J.C. (1992). Expression of the period clock gene within different cell types in the brain of Drosophila adults and mosaic analysis of these cells' influence on circadian behavioral rhythms. J. Neurosci. *12*, 3321–3349.

Frisch, B., Hardin, P.E., Hamblen-Coyle, M.J., Rosbash, M., and Hall, J.C. (1994). A promoterless period gene mediates behavioral rhythmicity and cyclical per expression in a restricted subset of the Drosophila nervous system. Neuron *12*, 555–570.

Gentile, C., Sehadova, H., Simoni, A., Chen, C., and Stanewsky, R. (2013). Cryptochrome antagonizes synchronization of Drosophila's circadian clock to temperature cycles. Curr. Biol. *23*, 185–195.

Glaser, F.T., and Stanewsky, R. (2005). Temperature synchronization of the Drosophila circadian clock. Curr. Biol. *15*, 1352–1363.

Helfrich-Förster, C., Yoshii, T., Wülbeck, C., Grieshaber, E., Rieger, D., Bachleitner, W., Cusamano, P., and Rouyer, F. (2007). The lateral and dorsal neurons of Drosophila melanogaster: New insights about their morphology and function. Cold Spring Harb. Symp. Quant. Biol. *72*, 517–525.

Körding, K.P., Beierholm, U., Ma, W.J., Quartz, S., Tenenbaum, J.B., and Shams, L. (2007). Causal inference in multisensory perception. PLoS ONE *2*, e943.

Levine, J.D., Funes, P., Dowse, H.B., and Hall, J.C. (2002a). Signal analysis of behavioral and molecular cycles. BMC Neurosci. *3*, 1.

Levine, J., Funes, P., Dowse, H., and Hall, J. (2002b). Resetting the circadian clock by social experience in Drosophila melanogaster. Science *298*, 2010–2012.

Miyasako, Y., Umezaki, Y., and Tomioka, K. (2007). Separate sets of cerebral clock neurons are responsible for light and temperature entrainment of Drosophila circadian locomotor rhythms. J. Biol. Rhythms *22*, 115–126.

Nitabach, M.N., and Taghert, P.H. (2008). Organization of the Drosophila circadian control circuit. Curr. Biol. *18*, R84–R93.

Peschel, N., and Helfrich-Förster, C. (2011). Setting the clock—by nature: Circadian rhythm in the fruitfly Drosophila melanogaster. FEBS Lett. *585*, 1435–1442.

Plautz, J.D. (1997). Independent photoreceptive circadian clocks throughout Drosophila. Science *278*, 1632–1635.

Renn, S.C., Park, J.H., Rosbash, M., Hall, J.C., and Taghert, P.H. (1999). A pdf neuropeptide gene mutation and ablation of PDF neurons each cause severe abnormalities of behavioral circadian rhythms in Drosophila. Cell *99*, 791–802.

Rieger, D., Shafer, O.T., Tomioka, K., and Helfrich-Förster, C. (2006). Functional analysis of circadian pacemaker neurons in Drosophila melanogaster. J. Neurosci. *26*, 2531–2543.

Roenneberg, T., and Merrow, M. (2016). The circadian clock and human health. Curr. Biol. *26*, R432–R443.

Sayeed, O., and Benzer, S. (1996). Behavioral genetics of thermosensation and hygrosensation in Drosophila. Proc. Natl. Acad. Sci. USA *93*, 6079–6084.

Sehadova, H., Glaser, F.T., Gentile, C., Simoni, A., Giesecke, A., Albert, J.T., and Stanewsky, R. (2009). Temperature entrainment of Drosophila's circadian clock involves the gene nocte and signaling from peripheral sensory tissues to the brain. Neuron *64*, 251–266.

Simoni, A., Wolfgang, W., Topping, M.P., Kavlie, R.G., Stanewsky, R., and Albert, J.T. (2014). A mechanosensory pathway to the Drosophila circadian clock. Science *343*, 525–528.

Stanewsky, R., Kaneko, M., Emery, P., Beretta, B., Wager-Smith, K., Kay, S.A., Rosbash, M., and Hall, J.C. (1998). The cryb mutation identifies cryptochrome as a circadian photoreceptor in Drosophila. Cell *95*, 681–692.

Vanin, S., Bhutani, S., Montelli, S., Menegazzi, P., Green, E.W., Pegoraro, M., Sandrelli, F., Costa, R., and Kyriacou, C.P. (2012). Unexpected features of Drosophila circadian behavioural rhythms under natural conditions. Nature *484*, 371–375.

Wheeler, D.A., Hamblen-Coyle, M.J., Dushay, M.S., and Hall, J.C. (1993). Behavior in light-dark cycles of Drosophila mutants that are arrhythmic, blind, or both. J. Biol. Rhythms *8*, 67–94.

Yao, Z., and Shafer, O.T. (2014). The Drosophila circadian clock is a variably coupled network of multiple peptidergic units. Science *343*, 1516–1520.

Yoshii, T., Vanin, S., Costa, R., and Helfrich-Förster, C. (2009). Synergic entrainment of Drosophila's circadian clock by light and temperature. J. Biol. Rhythms *24*, 452–464.

Yoshii, T., Hermann, C., and Helfrich-Förster, C. (2010). Cryptochrome-positive and -negative clock neurons in Drosophila entrain differentially to light and temperature. J. Biol. Rhythms *25*, 387–398.

Yoshii, T., Hermann-Luibl, C., and Helfrich-Förster, C. (2015). Circadian light-input pathways in Drosophila. Commun. Integr. Biol. *9*, e1102805.

Zerr, D.M., Hall, J.C., Rosbash, M., and Siwicki, K.K. (1990). Circadian fluctuations of period protein immunoreactivity in the CNS and the visual system of Drosophila. J. Neurosci. *10*, 2749–2762.

Cell Reports, Volume *17*

Supplemental Information

Sensory Conflict Disrupts Activity of the *Drosophila* Circadian Network

Ross E.F. Harper, Peter Dayan, Joerg T. Albert, and Ralf Stanewsky

Figure S1 (Related to Figure 1)

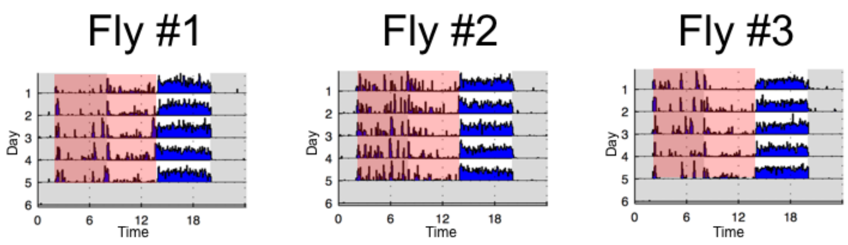

Figure S1. Novel P Behaviour Observed in Individual Flies
Three representative activity traces taken from individual CantonS flies during 6h misalignment of LD and TC (16:26°C). Red filter denotes warm phase; white background denotes light phase.

Figure S2 (Related to Figures 1 and 3)

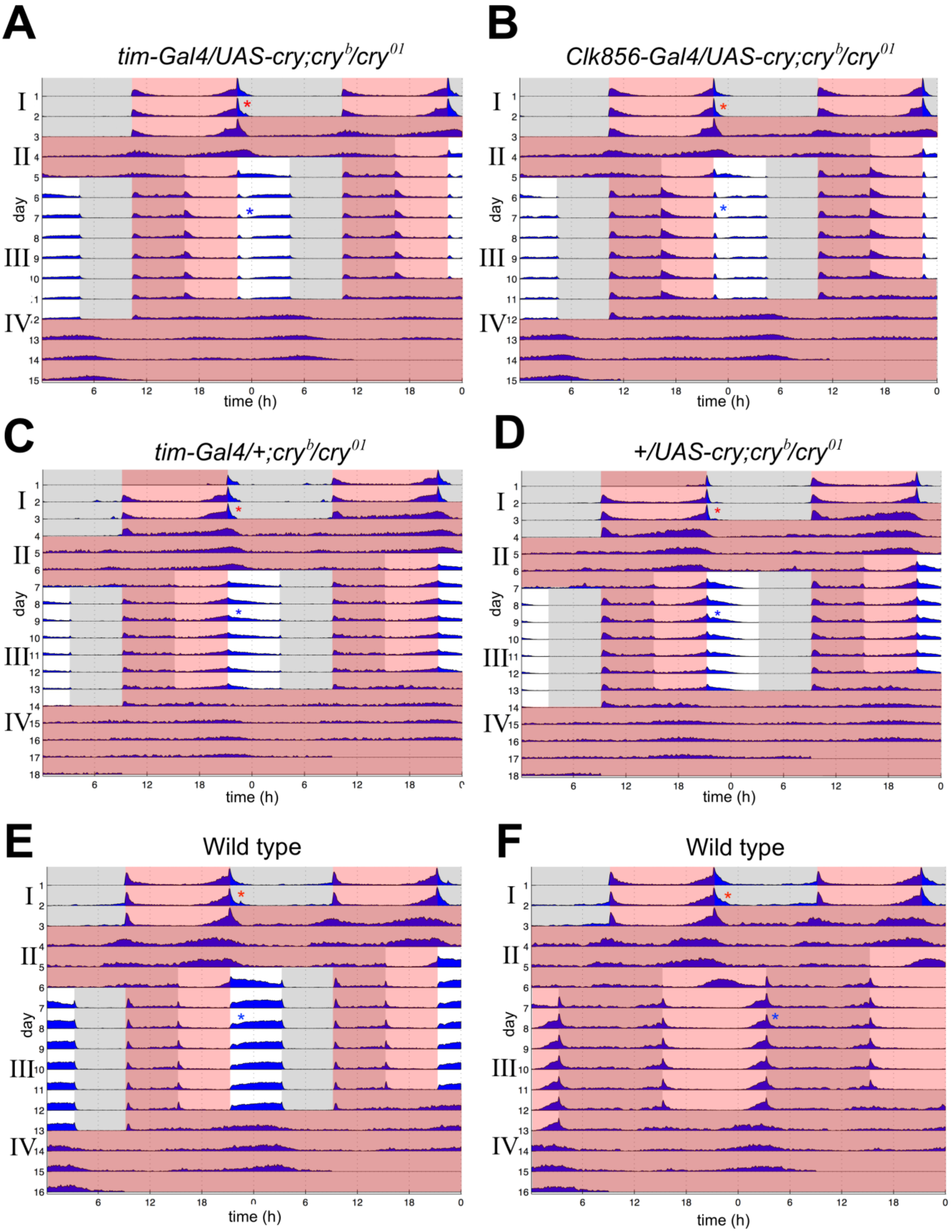

Figure S2. Circadian Locomotor Behavior During Sensory Conflict

Average actograms of (A) *cry* mutant rescue using a tim-Gal4 driver ($n = 32$), (B) *cry* mutant rescue using a *Clk856-Gal4* driver($n = 33$), (C*) tim-Gal4/+;cry^{b}/cry^{01}* control ($n = 12$), (D) *+/UAS-cry;cry^{b}/cry^{01}*control ($n = 21$), (E) Wild type repeat ($n = 38$), and (F) wild type during LD jet lag experiment (Part III comprises 6h delay of LD relative to Part I, at constant 26°C) ($n = 46$). Environmental conditions outlined in Figure 1. Red filter shows warm phase; white background shows light phase. Red asterisk denotes representative evening behaviour in Part I; blue asterisk denotes representative pseudo-evening behaviour in Part III.

Figure S3 (Related to Figure 3 and S2 E, F)

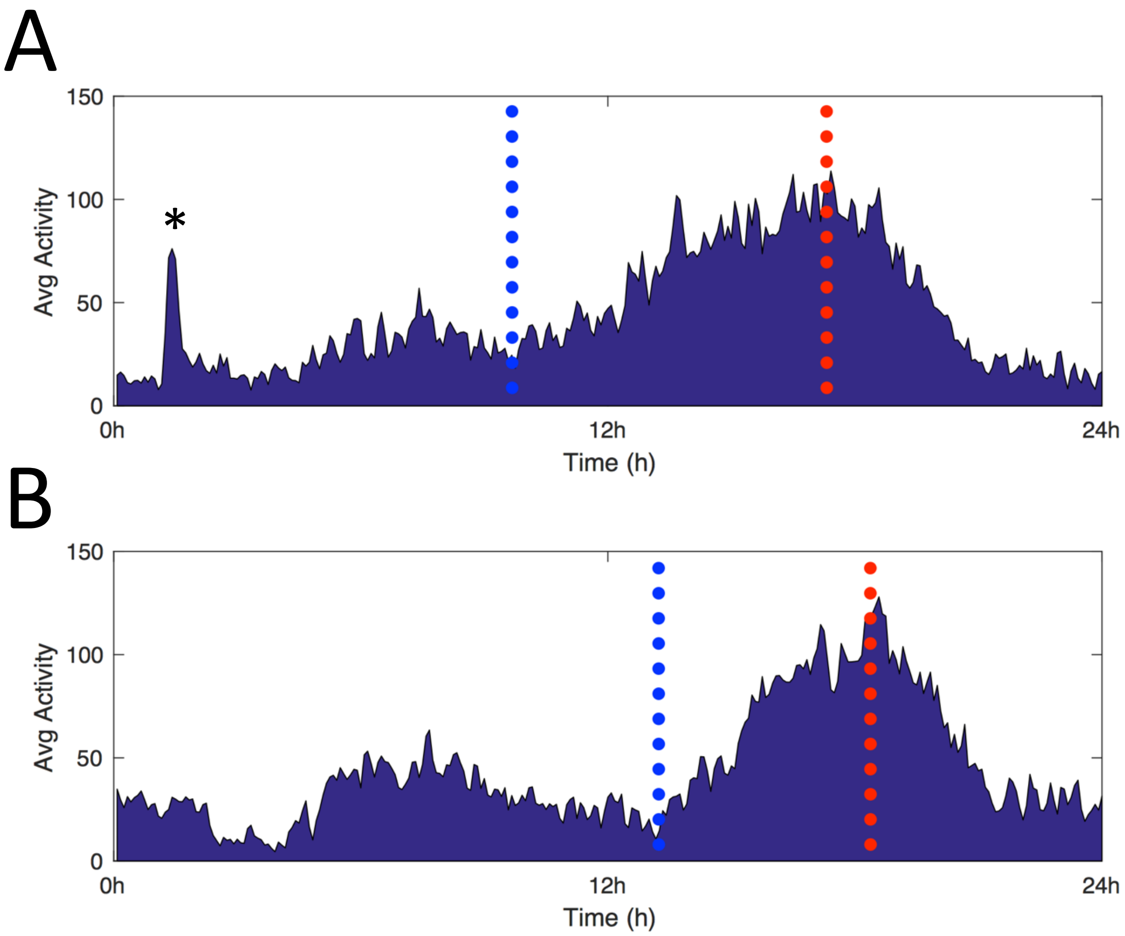

Figure S3. Average Fly Locomotor Activity During FR2
Average locomotor behaviour in wild type flies during the first 2 days of free run following 6h misalignment of LD:TC (A) and 6h LD shift in constant temperature (B). Average actograms covering the entire experiment can be seen in Figure S2 E,F. Red dashed line denotes peak phase; blue dashed line denotes activity onset. Black asterisk denotes startle behaviour elicited by onset of temperature at the beginning of free run following conflicting conditions.

Figure S4 (Related to Figure 4)

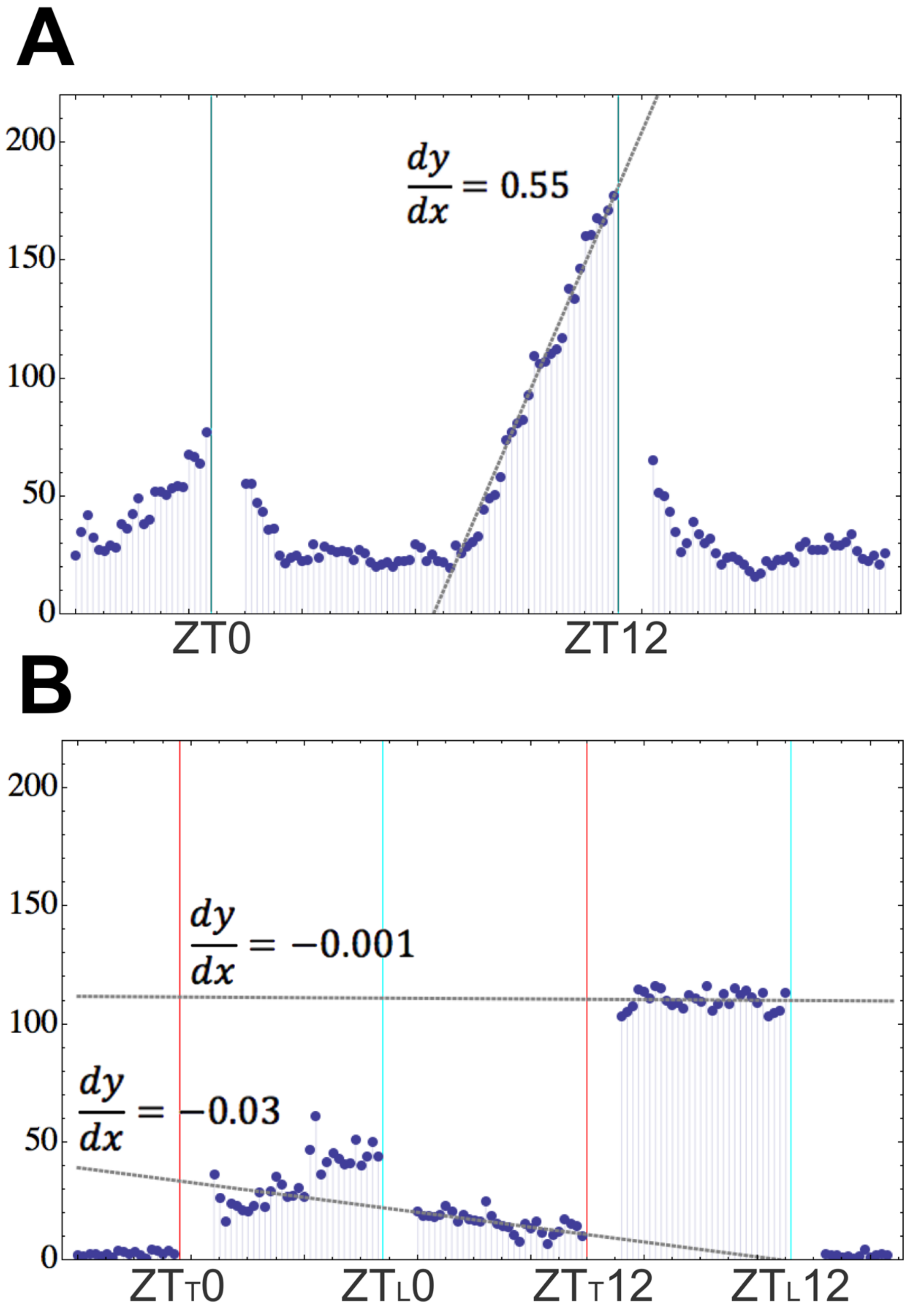

Figure S4. Evening Activity Gradient Quantification in wild type Flies
Linear regression is applied to the period of activity prior to Zeitgeber offset using MLE. Data points at, and 1hr after, the Zeitgeber transitions are excluded from the analysis. (A) When light and temperature are in-phase, $ZT_T = ZT_L$, and the linear increase of activity towards Zeitgeber offset is comparatively steep, showing typical locomotor entrainment. (B) When light and temperature are out-of-phase by 6hr, $ZT_T \neq ZT_L$, and thus we fit two linear regressions: one for the activity preceding temperature offset (red line), and one for the activity preceding light offset (cyan line).

Table S1 (Related to Figure 2). Statistics to accompany antibody staining data presented in Fig. 2.

One-Way ANOVA: P-values

**Part I**	**s-LN$_v$**	**l-LN$_v$**	**LN$_d$**	**DN$_3$**	**DN$_1$**	**DN$_2$**
Canton S	7.2×10^{-13}	6.6×10^{-27}	3.4×10^{-32}	5.8×10^{-37}	2.2×10^{-44}	2.9×10^{-21}
w;cry^{02}	2.1×10^{-13}	2.2×10^{-13}	1.7×10^{-22}	3.7×10^{-14}	6.9×10^{-29}	5.1×10^{-21}
**Part III**	**s-LN$_v$**	**l-LN$_v$**	**LN$_d$**	**DN$_3$**	**DN$_1$**	**DN$_2$**
Canton S	3.3×10^{-17}	5.0×10^{-16}	5.9×10^{-14}	1.5×10^{-13}	5.2×10^{-18}	3.4×10^{-7}
w;cry^{02}	3.8×10^{-17}	3.7×10^{-11}	3.4×10^{-19}	9.9×10^{-30}	5.9×10^{-21}	3.7×10^{-16}

Two-tailed T-Test Between ZT$_T$21 (ZT$_L$15) and ZT$_T$3 (ZT$_L$21) in Sensory Conflict: P-values

	**s-LN$_v$**	**l-LN$_v$**	**LN$_d$**	**DN$_3$**	**DN$_1$**	**DN$_2$**
CantonS	1.4×10^{-14}	1.2×10^{-9}	5.8×10^{-12}	5.0×10^{-5}	1.6×10^{-3}	1.4×10^{-4}
w;cry^{02}	1.4×10^{-3}	1.8×10^{-4}	5.0×10^{-7}	3.0×10^{-16}	1.4×10^{-5}	1.5×10^{-6}

N numbers (brain hemispheres)

	**Part I**	**s-LN$_v$**	**l-LN$_v$**	**LN$_d$**	**DN$_3$**	**DN$_1$**	**DN$_2$**
ZT$_T$21	Canton S	19	19	20	21	21	21
	w;cry^{02}	11	11	12	11	12	12
ZT$_T$3	Canton S	25	23	26	24	25	21
	w;cry^{02}	18	18	18	18	18	16
ZT$_T$9	Canton S	18	18	18	18	18	13
	w;cry^{02}	23	23	23	24	24	24
ZT$_T$15	Canton S	21	22	23	24	23	21
	w;cry^{02}	12	12	12	12	12	12
	**Part III**	**s-LN$_v$**	**l-LN$_v$**	**LN$_d$**	**DN$_3$**	**DN$_1$**	**DN$_2$**
ZT$_T$21	Canton S	33	34	34	34	32	31
	w;cry^{02}	19	20	20	14	14	16
ZT$_T$3	Canton S	33	32	35	35	34	26
	w;cry^{02}	25	26	26	25	24	22
ZT$_T$9	Canton S	18	18	17	19	20	09
	w;cry^{02}	23	24	24	24	24	24
ZT$_T$15	Canton S	28	28	32	32	32	32
	w;cry^{02}	16	16	16	17	16	16

Table S2. (Related to Figure 3) Quantification of free running activity rhythms in Part IV of the Experimental Regime

	τ(h)	RS	% Rhythmic	n
6h Conflict	24.6 ± 0.10	6.1 ± 0.2	100	38
LD Shift	24.3 ± 0.12	5.7 ± 0.19	95.7	44

Free running period values their significance (RS values) were determined as described in Supplemental Experimental Procedures. Period values associated with RS values ≥ 1.5 were considered rhythmic (Levine et al 2002)

Supplemental Experimental Procedures

Fly Strains

Canton S flies were used as wild type flies. Cryptochrome mutants were *w;cry*^{02} and *w;cry*^{01}*/cry*^{b} (Dolezelova et al., 2007; Stanewsky et al., 1998). For rescue experiments, *Clk856-gal4* (Gummadova et al., 2009), and *tim-gal4:27* (Kaneko and Hall, 2000), were crossed into a homozygous mutant *cry*^{b} background (Stanewsky et al., 1998) using appropriate balancer chromosomes and dominant markers. These *gal4* driver lines were then crossed to homozygous *cry*^{01} flies carrying *UAS-cry24.5* on chromosome *2* (Emery et al., 1998). F1 *Clk856-gal4/* or *tim-gal4:27/UAS-cry24.5, cry*^{b}*/cry*^{01} males were analyzed behaviorally as described below. Flies were reared under LD 12:12 cycles on *Drosophila* medium (0.8% agar, 2.2% sugar-beet syrup, 8.0% malt extract, 1.8% yeast, 1.0% soy flour, 8.0% corn flour, and 0.3% hydroxybenzoic acid) at 25°C and 60% humidity. Only male flies at an age of 3 to 6 days were used in experiments.

Activity Monitoring

Flies were individually placed into small glass recording tubes containing 5% sucrose and 2% agar medium, which occupied approximately one third of the tube. These tubes were then loaded into MB5 activity monitors (Trikinetics, Waltham, USA), with nine infrared beam detectors separated by 3mm directed at each activity tube. An interruption of the infrared light beam by the movement of a fly produced a signal, which was then recorded by a microprocessor. The number of beam breaks was recorded for each fly in 5-minute time bins and summed into bin counts. Thus, 12 activity scans were obtained for each fly per hour.

Monitors were placed in light- and temperature-controllable incubators (Percival) for the duration of the experiments. 12:12 LD was generated through square wave transition

between ~2500 and 0 lux respectively. 12:12 TC was achieved through transitions between 26°C (ON) and 16°C (OFF) occurring over ~10min. Environmental conditions were recorded with an environmental monitor placed inside the incubator. These were checked to validate scheduled conditions. (Details of specific experimental designs described at relevant points in Results).

Data Analysis

Activity of individual flies and average activity of the population were plotted as double actograms using the Matlab Flytoolbox library. Period length and proportion of rhythmic animals during free-running conditions, were calculated using autocorrelation in the Matlab Flytoolbox library (Levine et al., 2002). The autocorrelation output 'Rhythms Strength' (RS) serves as an estimate of the rhythm strength associated with each period value. In this study flies with RS values ≥ 1.5 were considered to be rhythmic (Levine et al., 2002, Table S2). To determine and compare the phase of the activity peaks during the two free-run parts of the experiment, circular statistics and phase plots using the same Matlab Flytoolbox library were used (Levine et al., 2002). All other analysis was carried out in Wolfram Mathematica using bespoke programs written for the purposes of this study (details of which are described in relevant sections of this report).

Quantification of Entrained Behavior

Analysis of locomotor behavior under entrained conditions is inherently challenging as observed behavior must be a result of both circadian drive and direct sensory effects (e.g. startle behavior and masking). It is common within the field to assess the anticipatory behavior prior to Zeitgeber offset - the so-called 'evening activity'. A simple measure of this evening behavior is a linear increase of activity prior to Zeitgeber offset.With this in mind, maximum likelihood estimation was used to best fit the linear regression, $y = a + bx$, to the

activity bout immediately prior to both light and temperature evening (Figure S3). The regression analysis was applied only to data points that displayed linearity preceding offset of the Zeitgeber in question. In cases where Zeitgeber offset for light and temperature were in close proximity, care was taken not to include evening activity for one stimulus in the analysis for the second. Thus, during very small or very large conflicts, fewer data points were available for fitting the later Zeitgeber, which translated into larger confidence intervals for these time points.

Immunostaining and Quantification

Flies collected at four time points during the in-phase and out-of-phase conditions (corresponding to ZT3, ZT9, ZT15 and ZT21 of the in-phase condition) were fixed in 4% paraformaldehyde in 0.1M phosphate buffer (PB) with 0.1% Triton X-100 (PBS-T) for 2.5h at room temperature. Flies were then rinsed three times in PB, and the brains subsequently dissected in PB. Brains were then blocked in 5% normal goat serum (NGS) in 0.5% PBS-T at 4˚C for 36h before incubation in primary antibodies for 48h at 4˚C. Double staining was conducted with primary antibodies: rabbit anti-PER (1:1500) (Stanewsky et al., 1997), and mouse anti-PDF (1:500) (DSHB). Secondary fluorescence-conjugated antibodies were alexaFluor 488 and alexaFluor 647 (purchased from Thermo Fisher Scientific), and were both diluted 1:300 in 0.5% PBS-T. Secondary antibodies were applied after washing six times in 0.5% PBS-T. After incubation with secondary antibodies, the brains were washed six times in 0.5% PBS-T and mounted in Vectashield (Vector Labs) mounting medium. The fluorescence signals of the whole mount brains were visualized using a Leica SP8 laser scanning confocal microscope.

Staining was quantified as described previously (Rieger et al., 2006), and a final staining index was calculated for each cell group:

$$SI = (Group\ mean\ pixel\ intensity - background) \times \frac{number\ of\ stained\ cells}{maximum\ number\ of\ cells\ in\ group}$$

The maximum number of cells for the different neuronal groups was as follows:

s-LN_v, 4; l-LN_v, 5; LN_d 7; DN_1, 17; DN_2, 2. Owing to the large number of DN_3 neurons, SI for this subgroup was calculated as $group\ mean\ pixel\ intensity - background$.

Statistical Methods

All statistical methods are described at relevant points in the text and supplemental information. In brief, phase comparisons of free-running activity rhythms were conducted using circular phase statistics using the Matlab Flytoolbox library (Levine et al., 2002). Here, activity data is smoothed using a low-pass filter, and average peak phase across two consecutive days is calculated for each fly. The results are then plotted in polar coordinates, and a dispersion test used to determine whether the two distributions (FR1 and FR2) differ significantly in angular deviation from their respective means.

For analysis of immunostaining data, one-way ANOVA was used to examine an effect of ZT on PER staining intensity. In addition to this, two-tailed t-test was used to compare staining of neuronal subgroups between time points as shown in Figure 2 and Table S1.

Supplemental References

Dolezelova, E., Dolezel, D., Hall, J.C., 2007. Rhythm Defects Caused by Newly Engineered Null Mutations in Drosophila's cryptochrome Gene. Genetics 177, 329–345. doi:10.1534/genetics.107.076513

Emery, P., So, W. V, Kaneko, M., Hall, J.C., Rosbash, M., 1998. CRY, a Drosophila clock and light-regulated cryptochrome, is a major contributor to circadian rhythm resetting and photosensitivity. Cell 95, 669–679. doi:10.1016/S0092-8674(00)81637-2

Gummadova, J.O., Coutts, G.A., Glossop, N.R.J., 2009. Analysis of the Drosophila Clock promoter reveals heterogeneity in expression between subgroups of central oscillator cells and identifies a novel enhancer region. J. Biol. Rhythms 24, 353–367. doi:10.1177/0748730409343890

Kaneko, M., Hall, J.C., 2000. Neuroanatomy of cells expressing clock genes in Drosophila: Transgenic manipulation of the period and timeless genes to mark the perikarya of circadian pacemaker neurons and their projections. J. Comp. Neurol. 422, 66–94. doi:10.1002/(SICI)1096-9861(20000619)422:1<66::AID-CNE5>3.0.CO;2-2

Levine, J., Funes, P., Dowse, H., Hall, J., 2002. Signal analysis of behavioral and molecular cycles. BMC Neurosci. 3, 1. doi:10.1186/1471-2202-3-1

Rieger, D., Shafer, O.T., Tomioka, K., Helfrich-Förster, C., 2006. Functional analysis of circadian pacemaker neurons in Drosophila melanogaster. J. Neurosci. 26, 2531–43. doi:10.1523/JNEUROSCI.1234-05.2006

Stanewsky, R., Frisch, B., Brandes, C., Hamblen-Coyle, M.J., Rosbash, M., Hall, J.C., 1997. Temporal and spatial expression patterns of transgenes containing increasing amounts of the Drosophila clock gene period and a lacZ reporter: mapping elements of the PER protein involved in circadian cycling. J. Neurosci. 17, 676–696.

Stanewsky, R., Kaneko, M., Emery, P., 1998. The cryb Mutation Identifies Cryptochrome as a Circadian Photoreceptor in Drosophila. Cell 95, 681–692.
